# The lipoprotein Pal stabilises the bacterial outer membrane during constriction by a mobilisation-and-capture mechanism

**DOI:** 10.1038/s41467-020-15083-5

**Published:** 2020-03-11

**Authors:** Joanna Szczepaniak, Peter Holmes, Karthik Rajasekar, Renata Kaminska, Firdaus Samsudin, Patrick George Inns, Patrice Rassam, Syma Khalid, Seán M. Murray, Christina Redfield, Colin Kleanthous

**Affiliations:** 1 Department of Biochemistry, University of Oxford, Oxford, OX1 3QU UK; 2 Department of Chemistry, University of Southampton, University Road, Southampton, SO17 1BJ UK; 3 Max Planck Institute for Terrestrial Microbiology and LOEWE Centre for Synthetic Microbiology (SYNMIKRO), Karl-von-Frisch Strasse 16, 35043 Marburg, Germany; 4 Present Address: Department of Biochemistry, University of Alberta, Edmonton, AB T6G 2H7 Canada; 5 Present Address: Evotec SE, 112-114 Innovation Drive, Milton Park, Abingdon OX14 4RZ UK; 6 Present Address: Laboratoire de Bioimagerie et Pathologie, UMR 7021, CNRS, Université de Strasbourg, Faculté de pharmacie, 74 Route du Rhin, 67401 Illkirch, France

**Keywords:** Lipoproteins, Bacterial structural biology, Cellular microbiology

## Abstract

Coordination of outer membrane constriction with septation is critical to faithful division in Gram-negative bacteria and vital to the barrier function of the membrane. This coordination requires the recruitment of the peptidoglycan-binding outer-membrane lipoprotein Pal at division sites by the Tol system. Here, we show that Pal accumulation at *Escherichia coli* division sites is a consequence of three key functions of the Tol system. First, Tol mobilises Pal molecules in dividing cells, which otherwise diffuse very slowly due to their binding of the cell wall. Second, Tol actively captures mobilised Pal molecules and deposits them at the division septum. Third, the active capture mechanism is analogous to that used by the inner membrane protein TonB to dislodge the plug domains of outer membrane TonB-dependent nutrient transporters. We conclude that outer membrane constriction is coordinated with cell division by active mobilisation-and-capture of Pal at division septa by the Tol system.

## Abstract (web summary)

The lipoprotein Pal participates in the coordination of outer-membrane constriction with septation in Gram-negative bacteria. Here, the authors show that this coordination is mediated by active mobilisation-and-capture of Pal at division septa by the Tol system.

## Introduction

Cell division in Gram-negative bacteria is orchestrated by the FtsZ ring that localises to mid-cell and establishes a multiprotein divisome complex^1^. Cycles of septal peptidoglycan synthesis/hydrolysis by the divisome and FtsZ treadmilling drive constriction of the entire cell envelope^2^. While much is known about the components that constrict the inner membrane and remodel the cell wall during cell division, relatively little is known about how the outer membrane (OM) is invaginated^3^. The OM is essential in most Gram-negative bacteria^4,5^, where it serves as both a permeability barrier to exclude hydrophobic and hydrophilic compounds, including antibiotics such as vancomycin^6^, and contributes to the mechanical stiffness of the cell^4^. The OM is not energised and there is no ATP in the periplasm so active processes at the OM must be coupled either to ATP hydrolysis in the cytoplasm or the proton motive force (PMF) across the inner membrane^7–9^. Recently, Petiti et al.^10^ have suggested that the multiprotein Tol system (also known as Tol-Pal) constricts the OM by populating the division septum with Pal. However, no mechanism has been proposed. In the present work, through a combination of in vivo imaging, deletion analysis and mutagenesis, structure determination, biophysical measurements, mathematical modelling and molecular dynamics simulations, we demonstrate that the PMF is exploited by the Tol system to both mobilise Pal in the OM of dividing cells and to then capture these mobilised molecules at division sites. Mobilisation-and-capture circumvents Pal’s intrinsically low mobility in the OM and results in its slow accumulation at division sites, where it invaginates the OM through non-covalent interactions with newly formed septal peptidoglycan.

**Fig. 1:**
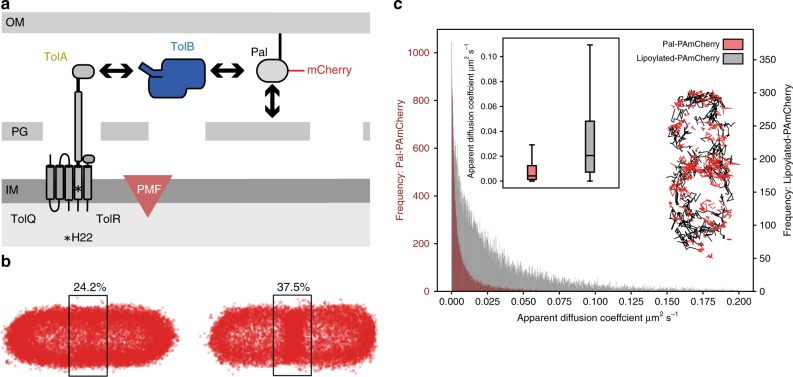
Interactions with the cell wall slow the diffusion of Pal in the outer membrane of *E. coli*. **a** Major components of the Tol system. The lipoprotein Pal, labelled with mCherry or PAmCherry (this work), non-covalently associates with the cell wall. Pal also binds to TolB through an interaction that is mutually exclusive of peptidoglycan binding. TolA spans the periplasm and is coupled to the PMF through interactions with two other inner membrane components, TolQ and TolR. The C-terminal domain of TolA interacts with the N-terminus of TolB (see below). Marked with an asterisk in TolA’s transmembrane helix is His22, which is essential for PMF coupling. **b** Pal-PAmCherry distribution in non-dividing (left-hand panel) and dividing (right-hand panel) cells. Panels show composite single-particle tracking data for 19,038 and 15,448 molecules in non-dividing and dividing cells, respectively. Data points represent the trajectory centroids of Pal-PAmCherry molecules normalised with respect to the cell. Pal has similarly low mobility in both cell types. Pal reorganises during cell division so that its concentration increases by ~55% at mid-cell relative to non-dividing cells. **c** Main panel, histogram showing apparent diffusion coefficients (*D*_app_) for Pal-PAmCherry (red) and lipoylated-PAmCherry (grey), for clarity diffusion coefficients above 0.2 μm^2^ s^−1^ are not shown. Removing the peptidoglycan binding domain of Pal increases *D*_app_ at least fivefold (0.004 μm^2^ s^−1^, *n* = 22,794 tracks compared to 0.021 μm^2^ s^−1^, *n* = 30,640 tracks in SPT experiments) and mobility becomes less constrained. Insert: right, single-particle tracks of Pal-PAmCherry (red) and lipoylated-PAmCherry (grey) molecules normalised with respect to the cell, a selection of tracks with diffusion coefficients close to their respective medians are displayed. Insert: left, Box plots of Pal-PAmCherry and lipoylated-PAmCherry diffusion coefficients (outliers not shown) a two-sided Student’s *t* test with unequal variances indicated a *p* value of ≪0.0001. For Pal-PAmCherry the first, second and third quartiles are 0.001, 0.004 and 0.012 μm^2^ s^−1^, respectively. The whiskers represent the most extreme data points that lie within the third quartile +1.5× the interquartile range and the first quartile −1.5× the interquartile range: 0.029 and 1.5 × 10^−7^ μm^2^ s^−1^, respectively. For lipoylated-PAmCherry the first, second and third quartiles are 0.007, 0.021 and 0.048 μm^2^ s^−1^, respectively. The most extreme data points that lie within the third quartile +1.5× the interquartile range and the first quartile −1.5× the interquartile range are 0.109 and 1.5 × 10^−6^ μm^2^ s^−1^, respectively. Source data are provided as a Source Data file.

*tol* genes, which are found in most Gram-negative bacteria, were originally identified by Luria and co-workers in the 1960s through mutations that engendered *Escherichia coli* tolerance towards colicins and filamentous bacteriophages^11^. Concomitant with this tolerance is a pleiotropic OM instability phenotype that is manifested through cell filamentation and division defects, hypersensitivity towards detergents and bile salts and leakage of periplasmic contents. The Tol assembly is essential in bacteria expressing O-antigens, is a virulence factor in host−pathogen interactions^12–14^ and is implicated in biofilm formation^15^. The core components of Tol are three IM proteins, TolQ, TolR and TolA, periplasmic TolB and peptidoglycan-associated lipoprotein Pal in the inner leaflet of the OM (Fig. 1a)^3^. TolA in the inner membrane spans the periplasm and undergoes PMF-driven conformational changes by virtue of its interaction partners TolQ and TolR, which are homologues of the MotA and MotB stator proteins that drive rotation of the bacterial flagellum (Supplementary Fig. 1)^16^. Pal binds the *meso*-diaminopimelate (mDAP) sidechain of stem peptides within the peptidoglycan layer^17^, but this non-covalent contact is blocked by TolB that binds Pal with high affinity and occludes the mDAP binding site^18^.

The pleiotropic phenotypes of *tol* mutants have obscured past efforts to define the role of Tol in the cell envelope, explaining why several functions have previously been ascribed to this trans-envelope assembly^19,20^.

Through a multidisciplinary approach, we now define the molecular mode of action of Tol. We show that during cell division the Tol assembly exploits the PMF to mobilise Pal molecules from around the cell while simultaneously capturing them at the divisome in order to stabilise the OM.

## Results

### The Tol system causes a ~50% increase in Pal molecules at the divisome

The recruitment of Tol components to the divisome^21^ in conjunction with the PMF concentrates Pal at the septum (Supplementary Figs. 2 and 3)^10^. However, it has not been established what proportion of Pal molecules in the cell are mobilised for OM stabilisation at division sites. To determine quantitatively the number of Pal molecules this represents and probe their diffusion characteristics, we followed the mobility of Pal fused to photoactivatable mCherry (Pal-PAmCherry) in *E. coli* by fluorescence microscopy (Fig. 1b). Single-particle tracking (SPT) experiments revealed that on short time-scales (~400 ms), the mobility of Pal-PAmCherry in both dividing and non-dividing cells is slow and highly restricted (median apparent diffusion coefficient, ~0.004 µm^2^ s^−1^; median confinement radius, ~83 nm; median trajectory localisation uncertainty, 19.3 nm). Slow Pal diffusion was attributed to cell wall binding since removal of the peptidoglycan-binding domain (lipoylated-PAmCherry) resulted in faster diffusion in the OM (Fig. 1c). Our experiments show that Pal-PAmCherry molecules are distributed randomly in non-dividing cells but redistribute with the onset of division; in non-dividing cells, ~24.2% of Pal molecules are located at mid-cell whereas in dividing cells ~37.5% of Pal molecules are located at mid-cell (Fig. 1b). We conclude that notwithstanding Pal’s slow mobility in the OM the onset of division results in a ~55% increase in the number of Pal molecules at the divisome. We set out to determine the mechanism by which this global mobilisation and septal localisation of Pal occurs during cell division.

### Mobilisation-and-capture of Pal underpins its active deposition

**Fig. 2:**
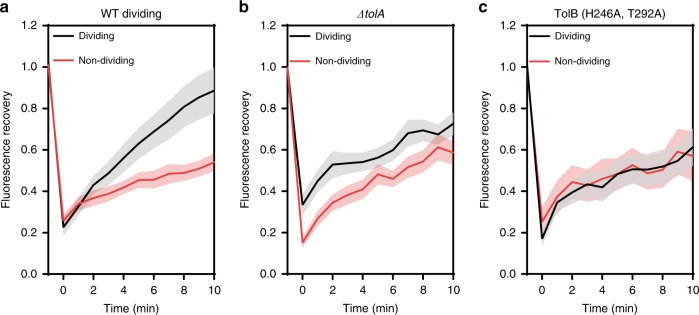
Fluorescence recovery after photobleaching (FRAP) data for wild-type and mutant *E. coli* cells. Fluorescence recovery curves of dividing and non-dividing cells after bleaching a rectangular area at the septum or mid-cell of non-dividing cells. All experiments were carried out at 37 °C. Recovery was monitored for 10 min post bleach. Recovery curves are an average from 30 cells, with bars representing 95% confidence interval (CI). **a** Wild-type dividing cells were able to slowly recover the fluorescence to higher level compared to non-dividing cells (~80% and ~60%, respectively). See text for details. **b** FRAP recovery curves of Pal-mCherry fluorescence in dividing and non-dividing cells of a *tolA* deletion strain. **c** FRAP recovery curves of Pal-mCherry fluorescence in dividing and non-dividing cells of a TolB mutant strain (TolB H246A T292A) where TolB is unable to bind Pal. In this instance, cephalexin (50 µg µl^−1^) was added to elongate non-dividing cells which were otherwise too small for FRAP analysis. Source data are provided as a Source Data file.

Suspecting the mobility of Pal in the OM must be even slower than that detected by SPT, we conducted fluorescence recovery after photobleaching (FRAP) experiments on *E. coli* cells expressing Pal-mCherry. We found that in non-dividing cells ~60% of the initial fluorescence at mid-cell recovered over a 10-min time-course which increased to ~80% in the septa of dividing cells (Fig. 2a). The very slow nature of this diffusion raised concerns that protein biogenesis and even reactivation of bleached fluorophores could be contributing to the recovery of fluorescence after photobleaching. Control experiments however indicated that these contributions were relatively minor (Supplementary Fig. 4). The acceleration in Pal diffusion evident during division required an intact Tol assembly since deletion of TolA or a mutant TolB that is unable to bind to Pal (TolB H246A T292A)^22^ both abolish this effect (Fig. 2b, c, respectively). Interestingly, the Pal mobility we measure is on a similar timescale to the elongation rate of an *E. coli* cell (~0.36 µm min^−1^, doubling time ~60 min) grown at 37 °C in defined media, as in the present experiments. We conclude that Pal diffuses very slowly in the OM yet still accumulates at mid-cell during division^23,24^.

**Fig. 3:**
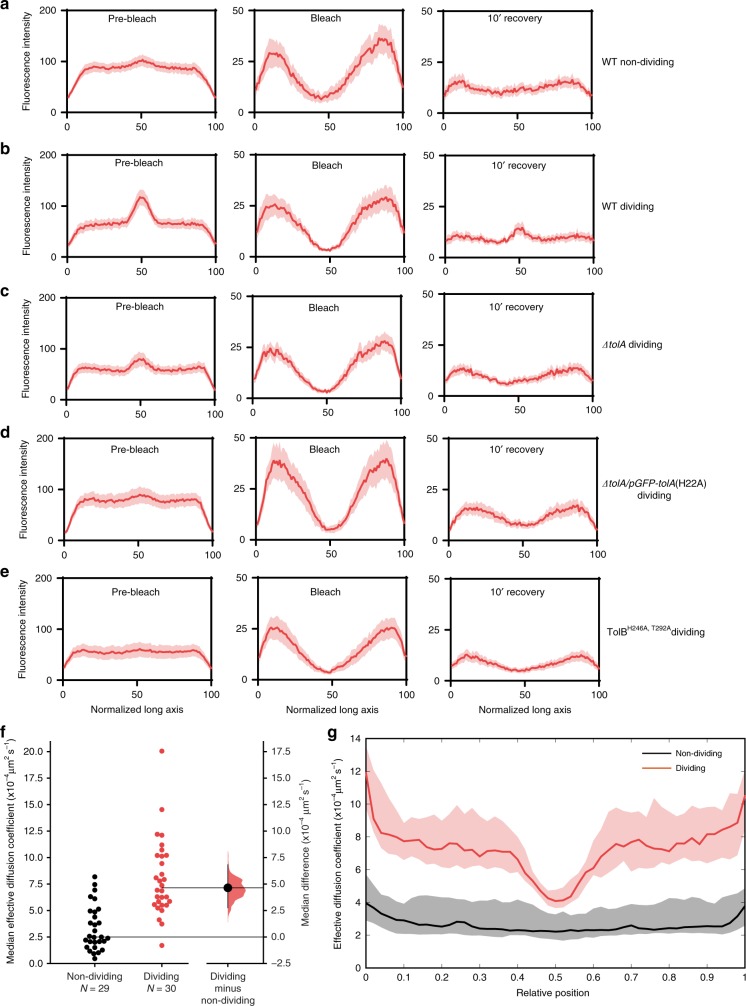
Pal dynamics in dividing and non-dividing *E. coli* cells. Panels **a**–**e** show longitudinal fluorescence distributions derived from FRAP data (Fig. 2) with 95% CI represented as shaded area. Left panels: cell before bleach. Middle panels: cells after bleach. Right panels: cells after 10 min recovery. **a** Wild-type non-dividing cells. **b** Wild-type dividing cells. **c** *tolA* deletion strain, dividing cells. **d** TolA H22A strain, dividing cells. **e** TolB H246A T292A strain, dividing cells. Only in the case of wild-type dividing cells does Pal-mCherry accumulation at the septum recover. **f** The effective diffusion coefficient (*D*_eff_) of Pal-mCherry in non-dividing and dividing cells (median across the cell-length) calculated from FRAP data as described in the text is shown in a Gardner−Altman estimation plot. Both groups are plotted on the left axes; the median difference is plotted on floating axes on the right as a bootstrap sampling distribution (5000 samples). The mean difference is depicted as a dot; the 95% confidence interval is indicated by the ends of the vertical error bar. The unpaired median difference between non-dividing and dividing cells is 4.65 with a 95.0% confidence interval range of [2.76, 6.83]. The two-sided *P* value of the Kruskal test is 4.1 × 10^−7^. The confidence interval is bias-corrected and accelerated. **g** The *D*_eff_ of Pal-mCherry as a function of relative cellular location in non-dividing and dividing cells obtained from FRAP data. Shown is the median across cells. Shading indicates the 95% confidence interval calculated using 1000 bootstrapped samples. Non-overlapping confidence intervals indicates difference are significant at the 95% confidence level. Source data are provided as a Source Data file.

To gain greater insight into the slow mobility of Pal and its dependence on the other components of the Tol system, we analysed the recovery FRAP curves across the longitudinal axis of wild-type and mutant cells. The data in Fig. 3a, b show that a significant fraction of photobleached Pal fluorescence re-emerges at the septum of dividing cells after 10 min whereas the same is not the case of the mid-cell position in non-dividing cells. Moreover, this recovery of septal Pal fluorescence is dependent on TolA, TolA coupling to the PMF and TolB binding to Pal (Fig. 3c–e, respectively). We conclude that the slow accumulation of Pal at division sites requires the TolQ-TolR-TolA IM complex, coupling of the complex to the PMF and TolB.

The incomplete recovery of Pal-mCherry fluorescence indicated that we would not be able to extract diffusion or binding rates using standard FRAP analysis because cells are growing and dividing over the same timescale as diffusion, plus the abundance of Pal-mCherry at mid-cell changes as division progresses (Fig. 1). We therefore developed a new mathematical method to extract effective diffusion coefficients (*D*_eff_) from the data. The method is based on fitting experimental spatial-temporal kymographs to numerically simulated kymographs, thereby making use of both the temporal and spatial information available (see Materials and Methods and Supplementary Fig. 5a). This kind of approach has been used previously^25^ to analyse a homogenously distributed protein (the 50 s ribosomal subunit). Here, we extend the technique to an inhomogeneous fluorescence distribution, which is fundamentally different to the homogeneous case. The resulting equation describing fluorescence recovery is the Fokker−Planck diffusion equation rather than the canonical Fickian diffusion equation. Additionally, the obtained effective diffusion coefficient, *D*_eff_, varies spatially across the cell. This is because *D*_eff_ depends on the binding and unbinding rates of Pal to and from the cell wall, one or both of which must necessarily vary spatially in order for Pal to be inhomogeneously distributed. More specifically, *D*_eff_ is the (spatially varying) proportion of mobile Pal molecules multiplied by the (usual) diffusion coefficient of mobile Pal. See Materials and Methods for further details. We used this method, which we call SpatialFRAP, to determine *D*_eff_ in individual dividing and non-dividing cells (Fig. 3f). We found the median *D*_eff_ across the cell to be very small in both cell types (on the order of 10^−4^ μm^2^ s^−1^), but significantly higher in dividing cells (difference of 4.65 μm^2^ s^−1^ × 10^−4^ μm^2^ s^−1^ with a 95.0% confidence interval of [2.76, 6.83] μm^2^ s^−1^).

We examined this difference in more detail by looking at how *D*_eff_ varies across the length of the cell (Fig. 3g). We found that *D*_eff_ in non-dividing cells was constant across the length of the cell but that dividing cells were characterised by a drop in *D*_eff_ from about 8 × 10^−4^ μm^2^ s^−1^ away from the septum to approximately 4 × 10^−4^ μm^2^ s^−1^, at the septum, closer to (but still statistically higher than) non-dividing cells (Fig. 3g). To control for the possibility that this was an artefact due to bleaching the septal region of the cell, we performed a similar experiment in which we bleached the pole of dividing cells (Supplementary Fig. 6) and found statistically similar results (within 95% confidence intervals). We also obtained *D*_eff_ for TolB H246A T292A and the *tolA* deletion strains to determine the contribution of the intact Tol system to this diffusion profile in both dividing and non-dividing cells. Both genetic backgrounds exhibited *D*_eff_ profiles and median values indistinguishable from non-dividing cells (Supplementary Fig. 5b, c). These FRAP data reaffirm the very slow diffusion of Pal in non-dividing cells, which, as shown above, is dominated by associations with the cell wall. Yet, remarkably, with the onset of division and localisation of the TolQ-TolR-TolA inner membrane complex to the divisome, the effective diffusion of Pal increases throughout the cell but primarily away from the septum. Blocking TolB binding to Pal or removing PMF-linked TolA abolished both the enhancement in *D*_eff_ and the accumulation of Pal at the septum. In conclusion, Pal mobility during division involves two distinct states; a septal state similar to that of a non-dividing cell in which diffusion is dominated by peptidoglycan binding, and, an alternate state, away from the division site where Pal mobility is enhanced. We next sought to determine what interactions within the Tol assembly could be responsible for generating these states.

### Structure of the TolA-TolB complex

**Fig. 4:**
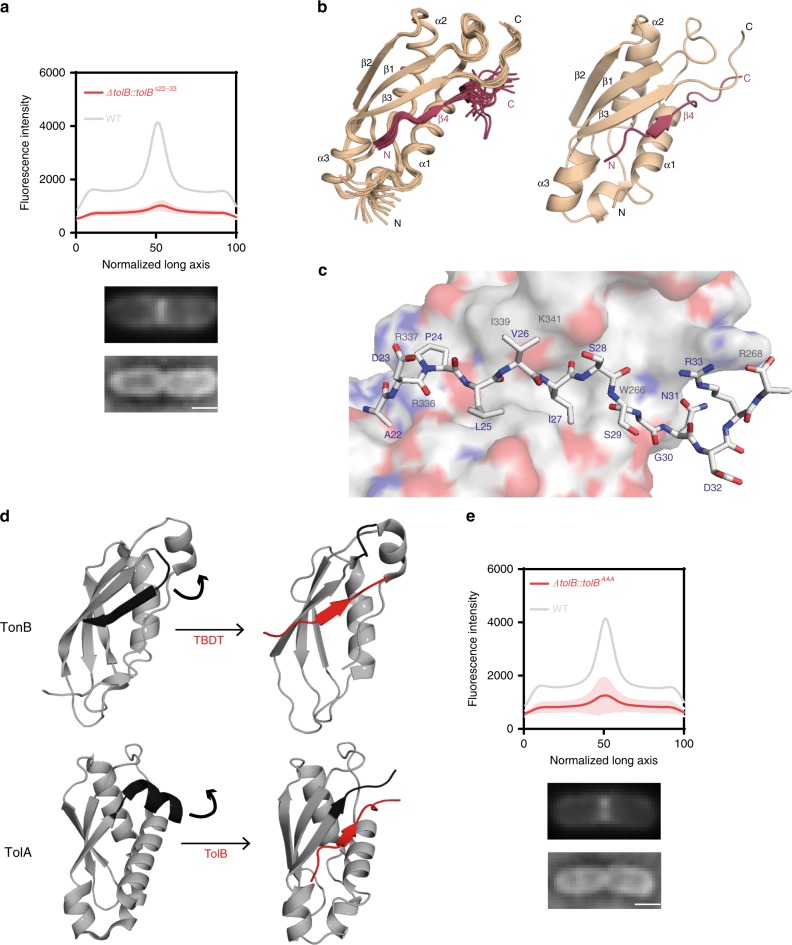
The structure of the TolA-TolB complex suggests a role in force transduction. **a** Average distribution of Pal-mCherry along the normalized *x*-axis of *E. coli* cells expressing mutant TolB from its native locus. Deletion of TolB’s N-terminus (residues 22−33) abolishes Pal-mCherry accumulation at the septum. The curve is an average from 30 cells, with shaded area representing standard deviation. Below: representative TIRFM and brightfield images of cells. Scale bar, 1 µm. **b** Solution-state NMR structure of the C-terminal domain of TolA (residues 224−347, beige) in complex with the N-terminus of TolB (the TolA box; residues 22ADPLVISSGNDRA34; red) from *P. aeruginosa*. See Supplementary Table 1 for full list of NMR restraints and refinement statistics. The first 23 residues of TolA (224−247) are unstructured in this complex and are not represented in the figure. Left-hand panel, overlay of the 20 lowest energy structures for the complex. Pairwise root mean squared deviation (RMSD) for the ensemble was 1.03 ± 0.12 Å. Right-hand panel, average ensemble structure for the complex. TolB^22–34^ binds by β-strand augmentation, forming a parallel β-strand with TolA. **c** TolB^22–34^, stick representation, binds to a cleft on the TolA surface. Hydrophobic residues in TolB play a prominent role in stabilising the complex (in particular Leu25, Val26 and Ile27). Source data are provided as a Source Data file. **d** β-strand augmentation is at the heart of both Ton and Tol complexes. The figure shows a comparison of a TonB-TBDT complex^71^ with TolA-TolB (present work). Complex formation in both cases requires a C-terminal element of secondary structure be displaced in order for a parallel β-strand to form. A structural overlay of the resulting complexes has an rmsd of 2.2 Å. **e** Alanine-substitution of the three key hydrophobic residues in *E. coli* TolB (Ile25Ala, Val26Ala, Ile27Ala; TolB^AAA^) abolishes Pal-mCherry accumulation at the septum of dividing cells. The fluorescence distribution shown is an average from 30 cells, with shaded area depicting standard deviation. Representative TIRFM and brightfield images of cells are shown below the fluorescence data. Scale bar, 1 µm. Source data are provided as a Source Data file.

Previous studies have shown that *E. coli* TolB interacts with both TolA and Pal whereas TolA only interacts with TolB (Fig. 1a)^18^. The disordered N-terminal 12 residues of TolB constitute the TolA binding site (*K*_d_ ~ 40 µM for the *E. coli* complex), since deletion of these residues abolishes TolA binding and results in a *tol* phenotype^18^. In the present work, we found that this deletion also abolished Pal-mCherry accumulation at division sites (Fig. 4a), demonstrating that the TolA-TolB complex is indeed essential for Pal deposition at septa.

To understand the structural importance of this complex, we determined the solution-state structure of the C-terminal domain of TolA bound to the N-terminus of TolB in the form of a peptide. Due to poor spectral resolution of the *E. coli* complex, however, we switched to the equivalent 12-kDa complex from *P. aeruginosa* (Supplementary Fig. 7). The structure of the *P. aeruginosa* TolA-TolB complex showed close structural similarity (2.2 Å rmsd) to complexes of *E. coli* TonB bound to the seven-residue TonB box sequence of TonB-dependent transporters (TBDTs) (Fig. 4b, c; Table 1). TonB is an inner membrane protein that extends through the periplasm so its C-terminal domain can activate import of iron siderophore complexes and vitamins through the plug domains that occlude TBDTs in the OM (Supplementary Fig. 1). The C-terminal domains of TonB and TolA share little sequence identity (<20%) but are structural homologues^26^. TolA-TolB and TonB-TBDT complexes are both formed by parallel β-strand augmentation in which a C-terminal element of secondary structure (a β-strand in TonB, an α-helix in TolA) is displaced in order to expose a peptidic binding site (Fig. 4d). In both cases, complexes of low affinity result, typically with equilibrium dissociation constants (*K*_d_) of 40–250 μM (Supplementary Fig. 7)^27^.

**Table 1:** NMR structure calculation statistics.

NOE-derived distance restraints	
Total	2901
Total unambiguous	2767
Intraresidue	1337
Interresidue	
Sequential (|*i*−*j*| = 1)	588
Short range (|*i*−*j*| = 2−3	283
Medium range (|*i*−*j*| = 4−5	82
Long range (|*i*−*j*|) > 5	399
Inter-chain	78
Total ambiguous	134
Hydrogen bond restraints	
TolA (intra)	27
TolA-TolB (inter)	4
Dihedral angle restraints	
TolA (*ϕ*/*ψ*))	69/69
TolBp (*ϕ*/*ψ*))	7/7
Residual dipolar couplings (RDCs)	
TolA	78
Structure statistics	
Number of restraint violations (mean ± s.d.)	
Distance restraint violations > 0.5 Å	0.95 ± 1.16
Dihedral angle violations > 5°	2.15 ± 0.57
RDC violations > 3 Hz	2.05 ± 0.97
RMSD from experimental restraints (mean ± s.d.)	
Distance restraints (Å)	0.057 ± 0.002
Dihedral angles (°)	1.049 ± 0.136
RDC restraints (Hz)	1.414 ± 0.050
RMSD from idealised geometry (mean ± s.d.)	
Bond lengths (Å)	0.0114 ± 0.0002
Bond angles (°)	1.044 ± 0.016
Impropers (°)	2.379 ± 0.086
Ramachandran analysis^a^ (%)	
Residues in most favoured regions	90.4
Residues in additional allowed regions	9.5
Residues in generously allowed regions	0.2
Residues in disallowed regions	0.0
Average pairwise RMSD^a,b^ (Å)	
Backbone (N, CA, C)	0.37 ± 0.06
All heavy atoms	1.03 ± 0.12

^a^Statistics applied to residues 3−9 and 254−340.

^b^Pairwise RMSD calculated for ensemble of 20 lowest energy structures.

The TolA-TolB complex buries 1519 Å^2^ accessible surface area, involves nine TolB amino acids and is stabilized by several hydrophobic interactions. The side-chains of three TolB amino acids, Leu25, Val26, Ile27, at the centre of the binding site replace hydrophobic contacts lost from the core of TolA as a result of its C-terminal α-helix being displaced (Fig. 4c). Displacement of this α-helix accounts for the slow association rate of the complex and for the slow chemical exchange dynamics evident in solution NMR experiments (Supplementary Figs. 7b, c and 8a–c). The importance of the stabilising hydrophobic contacts was demonstrated through single and multiple alanine substitutions all of which yielded *tol* phenotypes in vivo when mutated in *E. coli* TolB (equivalent residues Ile25, Val26, Ile27) and abolished *P. aeruginosa* TolB binding to TolA in vitro (Supplementary Fig. 8d, e). Moreover, TolB alanine mutations at these hydrophobic residues blocked Pal-mCherry accumulation at *E. coli* division sites (Fig. 4e), but left TolA localisation unaffected (Supplementary Fig. 8f). In conclusion, our data show that the same β-strand augmentation mechanism underpins formation of TolA-TolB and TonB-TBDT complexes.

### Molecular dynamics simulations suggest the TolB-Pal complex is force-labile

TonB-TBDT complexes convert the PMF into a mechanical force that displaces the plug domains of TBDTs in the OM^28^. It is reasonable to assume that TolA-TolB also transduces the force of the PMF into mechanical force. We postulate that this force is used to dissociate the TolB-Pal complex at the OM, releasing TolB to be recycled so that more Pal molecules can be mobilised for relocation to the division site. We next addressed the question of whether TolB-Pal complexes are susceptible to the application of force. We conducted steered molecular dynamics (MD) simulations in which *E. coli* TolB was pulled away from lipoylated Pal embedded in a membrane. (Supplementary Fig. 9a–c). An important consideration in these simulations was the structural state of TolB’s N-terminus, the TolA binding site. In the unbound state the TolB N-terminus is disordered, which favours TolA binding^18,29^. When Pal binds, large-scale conformational changes are induced in TolB that sequester its N-terminus between its two constituent domains and diminish TolA binding. Nevertheless, TolB-Pal can still interact with TolA to form a ternary (TolA-TolB-Pal) complex^18^, indicative of the N-terminus of TolB becoming dislodged from its inter-domain binding site. We therefore conducted simulations on the TolB-Pal complex in which the N-terminus was either bound to TolB or dislodged as would be necessary if TolA were to exert force on the complex. The simulations revealed that the application of force to the complex induced a conformational change that caused dissociation of Pal from the C-terminal β-propeller domain of TolB. Moreover, the pull-force required to dissociate the TolB-Pal complex was greater when the N-terminus of TolB remained bound to the body of TolB. The same trend was observed in two sets of steered MD simulations using different pulling speeds. The simulations identified a network of inter-domain hydrogen bonds involving TolB His146, Asp150 and Thr165 that appeared to link force to the dissociation of Pal in the simulations. The importance of these conserved TolB residues to the functioning of the system in vivo was evaluated by alanine mutations all of which yielded *tol* phenotypes (Supplementary Fig. 9d, e). Hence, our MD simulations supported by mutational data are consistent with the involvement of force in the release of Pal from TolB-Pal complexes. In the Discussion, we outline a model that reconciles force-mediated dissociation of the TolB-Pal complex at the OM by TolA with the mobilisation of Pal and its deposition at dividing septa.

## Discussion

The main role of the Tol assembly in Gram-negative bacteria is to concentrate the lipoprotein Pal at the divisome in order to bind the underlying peptidoglycan and invaginate the OM^10,21^. However, the Tol assembly is also implicated in other functions such as phospholipid homeostasis and septal peptidoglycan remodelling^20,30^. The latter role in particular is likely linked to the primary function of the assembly. TolA controls the oligomeric status of CpoB/YbgF, which is expressed from the *tol* operon and regulates transpeptidation at the septum by the PBP1B-LpoB complex^30,31^. The Tol assembly therefore appears to be playing a dual yet interconnected role at the divisome, on the one hand controlling the degree of peptidoglycan crosslinking and, on the other, actively localizing Pal at the septum which only binds non-crosslinked peptidoglycan.

**Fig. 5:**
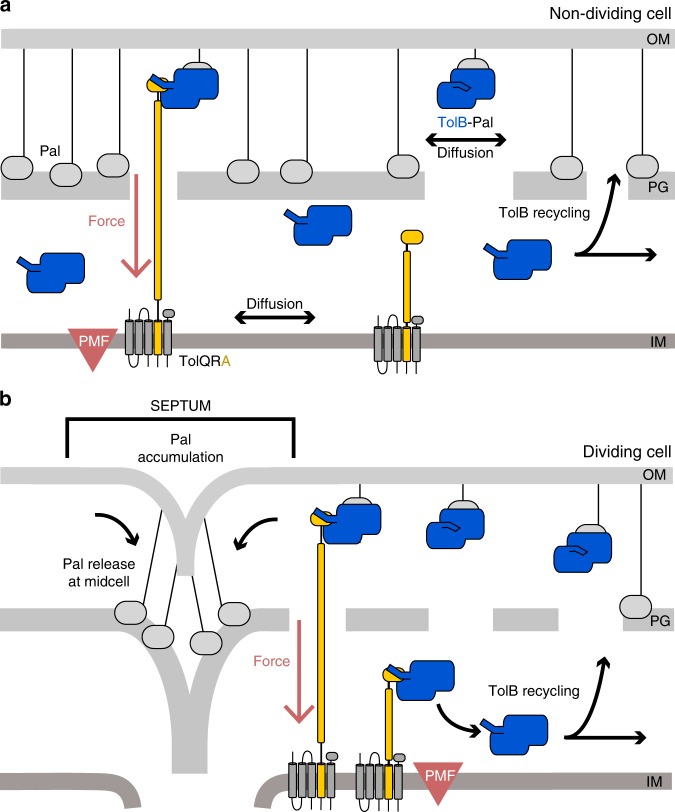
Mobilisation-and-capture of Pal by the Tol assembly drives active Pal accumulation at division sites. The following model views the periplasm as divided into two compartments separated by a porous peptidoglycan cell wall; the ‘outer periplasm’ is close to the outer membrane while the ‘inner periplasm’ is close to the inner membrane. **a** Non-dividing cells. In this state, TolQ-TolR-TolA is free to diffuse in the inner membrane. Periplasmic TolB enhances Pal mobility by blocking Pal’s association with peptidoglycan, but also marks the complex for active dissociation by TolQ-TolR-TolA via the N-terminus of TolB. We propose that TolA pulls TolB through the holes that exist in the peptidoglycan layer to the inner periplasm. Thereafter, TolB diffuses back through the peptidoglycan to the outer periplasm to repeat the process. Notwithstanding this recycling, however, Pal is predominantly free of TolB in non-dividing cells and therefore bound to the peptidoglycan layer, slowing its diffusion. **b** Dividing cells. The TolQ-TolR-TolA complex is recruited to the divisome which localizes its TolB capturing activity. This leads to an overall lowering of the level of TolB transduction through the peptidoglycan layer and consequently greater numbers of TolB molecules in the outer periplasm and as a result greater Pal mobility throughout the cell, except at the septum. Septal Pal is kept free of TolB by localized TolQ-TolR-TolA. As TolB-Pal complexes diffuse past the septum they are actively dissociated, releasing Pal. TolB is recycled to mobilise other Pal molecules away from the septum. Hence, only in dividing cells do the small number of TolBs enhance the mobility of a much larger population of Pal molecules, TolB acting essentially as an amplifier. The end result is that more and more Pal molecules become sequestered at the divisome where they stabilize the link between the OM and the underlying cell wall in daughter cells.

The present work demonstrates that a significant fraction of cellular Pal molecules become localised at the divisome to achieve OM invagination and lays out the basic mechanics by which the Tol assembly coordinates Pal localisation. An important consideration in understanding how the Tol assembly functions are the significant copy number differences of the individual proteins. Quantitative proteomics studies estimate ~60,000 copies of Pal in the OM of *E. coli* when grown in rich media, ~6000 copies of TolB and 500–2000 copies of the IM components TolA, TolQ and TolR^32^. The number of TolB molecules within the periplasm equates to a concentration of ~60 μM, which, given its high affinity for Pal (*K*_d_ ~ 30 nM), means that all TolB molecules will be in complex with Pal unless prevented from doing so. The affinity of Pal for peptidoglycan has not been reported but this is likely to be ~µM based on measurements of the affinity of the Pal-like domain of OmpA for peptidoglycan^33^. Below we integrate these copy number differences with our experimental data into a unifying model that explains how Tol exploits the PMF to cause Pal accumulation at division sites (Fig. 5). We first outline additional information and assumptions upon which this model is based.

First, we propose that the primary role of the PMF-coupled inner membrane complex of TolQ-TolR-TolA is to dissociate TolB-Pal at the OM by a process similar to that used by ExbB-ExbD-TonB to displace the plug domains of TBDTs^34^ (Supplementary Fig. 1). The PMF coupling mechanisms of the Ton and Tol machines in the inner membrane are known to be equivalent; indeed deletion mutants in one can be partially complemented by the other^19^. The present work demonstrates that the mechanical force generated by the PMF is transduced to the OM through structurally equivalent complexes, TonB-TBDT and TolA-TolB, respectively (Fig. 4d). Whereas TonB removes a soluble domain from within a TBDT, TolA removes a soluble protein, TolB, from its complex with Pal, most likely via a ternary TolA-TolB-Pal complex^18^. Our MD simulations, in conjunction with mutagenesis data, are consistent with the TolB-Pal complex being amenable to force-mediated dissociation. It has yet to be demonstrated however that PMF-coupled TolA in vivo can mechanically dissociate TolB-Pal complexes.

Second, Pal exists as two populations, a relatively immobile population bound to peptidoglycan and a smaller more mobile population bound to TolB. While we have not been able to detect the mobility of individual TolB-Pal molecules in dividing cells, our analysis indicates that at the population level the fraction of mobile Pal away from the mid-cell (leading to a higher *D*_eff_) is greater than in non-dividing cells (Fig. 3g). This implies that the concentration of mobile TolB-Pal complexes is also higher away from the septum in dividing versus non-dividing cells. Yet, TolB accumulates at the septum in dividing cells^10^. To resolve this apparent contradiction and explain the differential mobility of Pal we further propose that:

1. TolQ-TolR-TolA ‘pulls’ TolB through holes in the peptidoglycan layer^35^, from the ‘outer’ region of the periplasm close to the OM to the ‘inner’ periplasm thereby spatially separating TolB from Pal in the process. Precedent for such a trans-envelope pulling mechanism can be found in the TBDT literature. Transcription of TBDT genes is often activated by the cognate ligand binding to the TBDT in the OM, which relays a signal to the IM in a TonB-dependent manner^28^.
2. TolA-independent dissociation of the TolB-Pal complex is slow compared to the timescale of its diffusion, which is a reasonable assumption given that the half-life for dissociation of the TolB-Pal complex in vitro is ~2 min^36^. We now consider how the Tol system works in non-dividing and dividing cells (Fig. 5).

In non-dividing cells (Fig. 5a), TolQ-TolR-TolA in the inner membrane exhibits unrestricted Brownian motion^37^. We postulate that in this diffusive mode, and via coupling to the PMF, TolA continuously scans the OM for TolB-Pal complexes on which to pull. If a TolB-Pal complex is captured by circulating TolQ-TolR-TolA, then TolB is pulled from the outer to the inner periplasm thereby releasing Pal to bind peptidoglycan. TolB returns to the outer periplasm by eventually diffusing through holes in the peptidoglycan. Since TolB is actively translocated by TolQ-TolR-TolA in one direction, this will bias TolB to the inner periplasm. Our data suggest that in non-dividing cells this is the default position since the TolB H246A T292A mutant, which is unable to bind Pal, has no impact on Pal mobility (Supplementary Fig. 5c). Paradoxically then the PMF has the effect of slowing Pal mobility in non-dividing cells by ensuring Pal is always bound to peptidoglycan. Hence, in non-dividing cells the small number of TolB molecules relative to Pal (~10%) that could enhance Pal mobility in the OM are prevented from doing so because TolQ-TolR-TolA continually sequesters TolB to the inner periplasm.

In dividing cells (Fig. 5b), the TolQ-TolR-TolA assembly is recruited to the divisome^21,37^. An important consequence of this spatial localisation is that the assembly is now no longer available to dissociate TolB-Pal complexes anywhere other than the divisome. Hence, a TolB-Pal complex must diffuse further to interact with TolA, which could be half a cell-length away. This has the effect of reducing the translocation of TolB molecules from the outer-to-inner periplasm in dividing relative to non-dividing cells and therefore the abundance of TolB in the outer periplasm increases. This explains the observed differences in *D*_eff_ (Fig. 3g), which are indicative of increased Pal diffusion throughout the cell except at the septum. In other words, during division (and only during division), the small population of TolB molecules is harnessed to enhance the diffusion of a much larger population of Pal molecules in the cell. Once Pal is dissociated from TolB at the septum, it is free to bind newly formed peptidoglycan and so its levels accumulate. TolB on the other hand diffuses through the inner periplasm and eventually back through holes in the peptidoglycan layer to return to the outer periplasm where the cycle repeats. Effectively then, TolB acts as a TolA-powered conveyor belt for bringing Pal to the septum. This interpretation also explains the ‘action-at-a-distance’ observed on cellular Pal diffusion when TolQ-TolR-TolA becomes localised at the septum. Another consequence of this localisation is that Pal bound at the septum is kept free of TolB since it is continually dissociated by TolA, explaining why Pal at division sites has very similar diffusive characteristics as Pal in non-dividing cells.

There are individual elements of the mechanism we propose that have yet to be validated in vivo, such as the enhanced diffusion of Pal when bound to TolB, the displacement of Pal-TolB complexes by PMF-coupled TolA and the partitioning of TolB molecules between the compartments of the periplasm created by the cell wall. Collectively, however, our data support a mobilisation-and-capture mechanism for Pal accumulation at division sites that is driven by the entire Tol system (Fig. 5). The mechanism is an elegant solution to the problem of how to actively stabilize the OM when the membrane itself is not energized and neither is the protein doing the stabilising. An important outcome of this mechanism is that the mobility and localisation of Pal are both tuned to the division state of the cell.

## Materials and methods

### Strain and plasmid construction

Plasmid pREN126 (Table 2) encoding *E. coli* Pal-mCherry was generated by ligating *Xba*I/*Sal*I digest of *pal* gene PCR-amplified from MG1655 genomic DNA fused together^38^ with the mCherry gene optimised for *E. coli* expression into the plasmid pDOC-K. The strain RKCK8 (*pal::pal-GGGGS-mCherry*) (Table 3) was engineered using λ-Red recombination based on the gene-doctoring protocol^37,39^, all the consequent strains were constructed using the same method. QuickChange mutagenesis was used to introduce point mutations into genes where needed.

**Table 2:** Plasmids used in the study.

Plasmid	Genotype	Reference
pACBSCE	*Para* controlled λ-Red recombinase	^39^
pDOC-K	*bla* flanked by FLP	^39^
pCP20	*bla cat cI857 repA(ts) PR::flp*	^72^
pREN126	pDOC-K-336bp upstream *pal (ΔAgeI)-pal-GGGGS-mCherry (ΔAgeI)(ΔEcoRI)-bla-300* bp-downstream *pal*	This study
pNP4	pBR322-GFP-t*-tolA*	^21^
pREN88	pBR322-GFP-t*-tolA*(H22A)	^37^
pDAB17	pBAD24-*tolB*	^18^
pGP3	pBAD24-*tol-*HIS_6_	This study
pGP89	pBAD24p*tol*(H146A)-HIS_6_	This study
pGP90	pBAD24-*tol*(D150A)-HIS_6_	This study
pGP95	pBAD24-*tol*(T165A)-HIS_6_	This study
pREN71	pBAD24-*tol*(L25A)	This study
pREN72	pBAD24-*tol*(L25A, I27A)	This study
pREN70	pBAD24-*tol*(L25I, I27A)	This study
pREN73	pBAD24-*tol*(L25A, V26A, I27A)	This study
pREN28	pQE-Im9-FXa- *P. aeruginosa* TolA	This study

**Table 3:** Strains used in the study.

Strain name	Genotype	Reference
BL21 (DE3)	F- *ompT hsdSB(rB–, mB–) gal* dcm	^74^
DH5α	F- *endA1 glnV44 thi-1 recA1 relA1 gyrA96* deoR nupeptidoglycan Φ80dlacZΔM15 Δ(*lacZYA- argF*)U169, *hsdR17*(rK- mK+), λ–	^75^
BW25113	F-, Δ(*araD-araB*)*567* Δ(*rhaD-rhaB*)*568 ΔlacZ4787* (::rrnB-3) *hsdR514 rph-1*	^73^
JW0729-3	F-, *Δ(araD-araB)567, ΔlacZ4787(::rrnB-3), ΔtolA788::kan, λ-, rph-1, Δ(rhaD-rhaB)568, hsdR514*	^76^
JW5100-1	F-, *Δ(araD-araB)567, ΔlacZ4787(::rrnB-3), ΔtolB789::kan, λ-, rph-1, Δ(rhaD-rhaB)568, hsdR514*	^76^
JW0731-1	F-*, Δ(araD-araB)567, ΔlacZ4787(::rrnB-3), Δpal-790::kan, λ-, rph-1, Δ(rhaD-rhaB)568, hsdR514*	^76^
RKCK8	BW25113 *pal::pal-GGGGS-mCherry(ΔAgeI)(ΔEcoRI)-kan*	This study
JSCK1	JW0729-3 *Δkan::frt*	This study
RKCK10	JSCK1 Δ*pal::pal-GGGGS-mCherry(ΔAgeI)(ΔEcoRI)-kan*	This study
JSCK4	JW5100-1 *Δkan::frt*	This study
RKCK12	JSCK4 *pal::pal-GGGGS-mCherry(ΔAgeI)(ΔEcoRI)-kan*	This study
JSCK8	RKCK8 *Δkan::frt*	This study
RKCK13	JSCK8 *tolB::tolB*Δ22-33	This study
RKCK14	JSCK8 *tolB::tolB*(I25A V26A I27A)	This study
RKCK15	JSCK8 *tolB::tolB*(H246A T292A)	This study
RKCK16	BW25113 *pal::pal-GGGGS-PAmCherry*	This study
RKCK19	BW25113 *pal::pal(1-59)-PAmCherry*	This study

### Cell preparation for live microscopy

Overnight supplemented M9-glucose (2 mM MgSO_4_, 0.1 mM CaCl_2_, 0.4% (w/v) d-glucose) cultures were diluted in fresh medium with appropriate antibiotics and IPTG for strains expressing TolA from plasmids. Cultures were grown at 37 °C to OD_600_ 0.3. Cells were centrifuged at 7000 × *g* for 1 min, resuspended in 1 ml of fresh media and treated with CCCP (0.1 mM, RT for 5 min, Sigma #C2759) for PMF decoupling, sodium azide (50 µg ml^−1^, RT for 30 min, Sigma #S2002) for ATPase inhibition or chloramphenicol (30 µg ml^−1^, 37 °C for 30 min, Sigma #C0378) for translation inhibition. Agar pads were prepared by mixing supplemented M9-glucose medium with 1% agarose and pouring 200 µl into 1.5 × 1.6 gene frame (Thermo Scientific #AB0577) attached to the slide. For pad formation, the gene frame was sealed by a coverslip until agarose solidified. Where drug was used, such as chloramphenicol, they were incorporated into the pad. 5 µl Five microliters of cells was pipetted onto the agar pad, allowed to dry and sealed with a clean coverslip. For Pal-PAmCherry and lipoylated-PAmCherry SPT-PALM experiments, *E. coli* strains: RKCK16 and RKCK19 were used, respectively. Cells were grown to OD_600_ 0.3–0.6, a 500 µl aliquot was pelleted and resuspended in 40 µl M9-glucose supplemented with 30 µg ml^−1^ kanamycin. 7.2 µl of resuspended cells was loaded onto a 1% agarose pad using PBS as the diluent.

### TIRFM acquisition

Live cells were imaged using an Oxford NanoImager (ONI) superresolution microscope equipped with four laser lines (405/473/561/640 nm) and ×100 oil immersion objective (Olympus 1.49 NA). Pal-mCherry fluorescence images were acquired by scanning a 50 µm × 80 µm area with a 561 nm laser (0.2 mW) set at 49° incidence angle (100 ms exposition), resulting in a 512 × 1024 pixel image. For PAmCherry experiments, fluorophores were first activated by 1 s exposure to a 405 nm laser. Images were recorded by NimOS software associated with the ONI instrument. If not noted otherwise, each image was acquired as a 100- or 200-frame stack for brightfield and fluorescence channels, respectively. For analysis, images were stacked into composite images using average intensity as a projection type in ImageJ (version 1.52n).

### FRAP acquisition

Microscopy was performed on a Zeiss LSM 880/Axio Examiner Z1 motorised upright laser scanning microscope equipped with Argon multiline 458/488/514 nm (25 mW), and HeNe 561 nm (1 mW) and ×63 oil-immersion objective (Zeiss, NA 1.4) set to 37 °C. Cells were imaged by scanning the laser over a 13.5 × 13.5 μm area with the scan speed set to 8 or 16 and a digital zoom of ×10 or ×20, respectively. The diameter of the pinhole was 0.83 µm. Bleaching of the set region of interest (ROI) was performed on 20 × 50 or 30 × 80 pixel area (for ×10 or ×20 zoom, respectively) using 15 scan iterations and the corresponding laser power set to 100%. Two images were acquired before bleaching and ten images, with a time interval of 1 min, recorded after bleaching using an automatic time-course function. Instrument autofocus was used between images in reflection mode. All images were acquired using 2% maximum laser power. For all fluorescent images, corresponding differential interference contrast (DIC) images were recorded using transmitted light. All images were recorded using Zeiss Zen 2011 software.

### PALM-SPT acquisition

Pal-PAmCherry and lipoylated-PAmCherry SPT microscopy was conducted at 37 and 25 °C, respectively on an ONI inverted superresolution microscope. To image Pal-PAmCherry, an excitation laser of 561 nm was on for the entire duration of imaging at a power output of approximately 20–40 mW, an activation laser of 405 nm was manually pulsed at a power output of approximately 0.1–1 mW. For Pal-PAmCherry and lipoylated-PAmCherry SPT, 1000–14,000 frames were collected with an exposure time of 50 ms. For Pal-PamCherry, four repeats were acquired and for lipoylated-PAmCherry six repeats were acquired.

### SIM acquisition

Live bacteria were mounted between an agarose pad and a coverslip. Imaging was processed at room temperature with a ×60, NA 1.42 oil objective on a Deltavision OMX V3 Blaze (GE) equipped with a 488 laser. Image stacks of several μm thickness were taken with 0.125 μm *z*-steps and 15 images (three angles and five phases per angle) per *z*-section and a 3D-structured illumination with stripe separation of 213 nm. Laser power was set at 10% to scan the sample over 4 µm thickness (32 slices) without too much photobleaching. Image stacks were reconstructed using Deltavision softWoRx 6.1.1 software with a Wiener filter of 0.002 using wavelength-specific experimentally determined OTF functions (see ref. ^37^). Cells were automatically aligned using ImageJ software so the fluorescent ring appears as a vertical bar from the lateral projection of 3D-SIM imaging. Distribution of fluorescence intensity along the *x*-axis of bacteria was determined using a custom script implemented in MATLAB (version 2012a, MathWorks) (see ref. ^40^). In addition, with noise reduction through application of the built-in MATLAB medfilt2 median filter, raw images were initially thresholded over twofold the background intensity (optimised to provide a binary image highlighting specific signal from the septation ring). Intensity along the selected bacteria was measured 11 times between the bacterial end-points, each at a uniformly spaced offset from the bacterial long axis. The mean profile was calculated and normalized to the range 0–100 for comparison between bacteria. Diameter measurements correspond to the height of ring, calculated automatically through ImageJ software from each reconstructed and filtered image. A total of 30 cells were analysed per condition, in triplicate. All data were plotted in GraphPad software (Prism V).

### Image data analysis

All images were analysed using ImageJ software. Fluorescence distribution along *x*-axis of cells was determined by the plot profile function with a line width of 4 points for TIRFM images and 40 or 80 for FRAP images. Values were then normalized to 0−100 for comparison between cells in Excel. FRAP values were normalized using FRAPnorm plugin^37^. For all experiments, ~30 cells were analysed from at least three independent repeats per condition. All data were plotted in GraphPad Prism 8 software.

### SPT data analysis

The ImageJ plugin ThunderSTORM^41^ was used to localise single particles. The PSF Integrated Gaussian method in ThunderSTORM was used for sub-pixel localisation, to remove aberrations, localisations with photon offsets of <1 and sigma values <1 were filtered out. For fields of view that displayed significant XY drift, this was corrected using ThunderSTORM cross-correlation drift correction with five bins at a magnification of 5. Localisation information was imported into the ImageJ plugin TrackMate^42^, tracks between localisations were determined using a simple LAP tracker with a maximum linking distance of 250 nm and a gap-closing max frame gap of 0, trajectory information was exported. For each SPT experiment, a superresolution reconstruction of Pal-PAmCherry or lipoylated-PAmCherry distribution was generated from the localisations. With reference to the superresolution reconstruction, cells were manually segmented to generate a binary image, displaying cells in white and background in black. For Pal-PAmCherry SPT: cells were further separated into dividing and non-dividing categories dependent on the observation of a septum in the superresolution reconstruction. Custom Python scripts were developed to process the single-particle tracking data, with the following functionality: (1) Elimination of trajectories consisting of fewer than five consecutive localisations. (2) Elimination of trajectories not occurring within cells, with reference to the binary image. (3) Determination of diffusion coefficients. (4) Determination of the radius of the smallest circle that encapsulates all of the localisations in a trajectory. (5) Determine the mean uncertainty of the localisations in a trajectory. (6) Normalise the location of the centroid of each trajectory with respect to the cell.

The pixel coordinates of the binarised cells were identified and stored. The centroid of each trajectory was determined and the XY coordinates rounded to the nearest whole value; if the trajectory centroid occurred within the coordinates of a cell, they were retained, otherwise they were eliminated. Retained trajectories are referred to as “refined trajectories”, henceforth.

For refined trajectories, the mean squared displacement (MSD) was calculated at four lag times: 50, 100, 150 and 200 ms. The line of best fit of MSDs at these four lag times was used to identify the diffusion coefficient (analogous to the method used in ref. ^40^), following the equation:

$${\it{MSD}} = 4{\it{D}}\Delta {\it{t}},$$

where *D* is the two-dimensional diffusion coefficient. For trajectories in which the line of best fit of the MSD plot gave a negative value and hence a negative diffusion coefficient, these diffusion coefficients were eliminated.

For each refined trajectory, the centroid of the trajectory was determined. The radius of the smallest circle, centred at the centroid of the trajectory, which encapsulated all of the individual localisations of the trajectory was determined in nm.

From the binarised image, an ellipse was fitted to each cell, using the minor and major axis lengths and the angle of rotation of these ellipses, cells were normalized so that the long axis of the cell was represented using values ranging from 0 to 100 arbitrary units and the short axis of the cell was represented using values ranging from 0 to 50 arbitrary units. These transformations were applied to the centroid of each refined trajectory to determine the location of trajectories in a normalised cell. This normalisation was also applied to all of the localisations within entire trajectories to generate the normalised trajectory data displayed in Fig. 1c.

### Modelling Pal diffusion

We consider Pal as being in one of two states, depending on its binding to peptidoglycan. We assume that the diffusion of Pal when bound to peptidoglycan is very slow compared to when it is not bound to peptidoglycan so that we can set the bound diffusion constant to zero. We denote the concentrations (or molecular number) of free and bound Pal, as a function of position *x* along the long axis of the cell, by *u* = *u*(*x, t*) and *v* = *v*(*x, t*), respectively. Binding to, and unbinding from, peptidoglycan occur at rates *k*_on_(*x*) and *k*_off_(*x*), respectively, which can vary as a function of position. We then have the following equations:

$$\frac{{\partial u}}{{\partial t}} = D\frac{{\partial ^2u}}{{\partial x^2}} - k_{{\mathrm{on}}}\left( x \right)\,u + k_{{\mathrm{off}}}(x)\,v,$$

$$\frac{{\partial v}}{{\partial t}} = - k_{{\mathrm{on}}}\left( x \right)\,u + k_{{\mathrm{off}}}(x)\,v.$$

We further assume that the timescale of binding and unbinding is much shorter than that of (free) diffusion. This is the so-called effective diffusion regime^43,44^ and allows us to write $v\left( {x,t} \right) = \frac{{k_{{\mathrm{on}}}\left( x \right)}}{{k_{{\mathrm{off}}}\left( x \right)}}u(x,t)$ and thereby derive a single equation for the total concentration of Pal = *u* + *v*:

$$\frac{{\partial c}}{{\partial t}} = \frac{{\partial ^2}}{{\partial x^2}}\left( {D_{{\mathrm{eff}}}(x)\,c} \right),$$

where $D_{{\mathrm{{eff}}}}(x) = \frac{{k_{{\mathrm{off}}}(x)}}{{k_{{\mathrm{on}}}\left( x \right) + k_{{\mathrm{off}}}(x)}}D$ is the effective diffusion coefficient. Note that it can vary spatially according to the spatially dependent on- and off-rates. However, it has a simple interpretation; at each spatial location, it is the fraction of free molecules multiplied by the free diffusion coefficient.

The above equation for *c* was fitted to the experimental data as follows. We begin with the mean signal along the length of the cell on each frame. We corrected for background fluorescence by subtracting the mean background fluorescence outside the cells. We then binned the data in order to remove noise from over-sampling to an effective pixel size of 65.5 nm (WT and *tolA* mutants) or 52.4 nm (*tolB* mutant). We next removed the first and last two (binned) pixels to remove effects due to lower signal at the poles and finally we normalized the sum of the signal to 1. We then assume that Pal is at equilibrium at any given moment in time and take the pre-bleach frame as the equilibrium profile. The equation above, combined with vanishing flux boundary conditions, has an equilibrium profile that is, up to a constant, the reciprocal of *D*_eff_(*x*). The pre-bleach frame is therefore taken to specify, up to a constant, *D*_eff_(*x*). We determine the proportionality constant by fitting the solution to the above equation with the first post-bleach frame as the initial condition to the experimental post-bleach frames. Including the first post-bleach frame, we fit the model output to the data from six frames each separated in time by 2-min intervals (the final frame is 10 min post-bleach). We used the *pdepe* solver in Matlab to numerically solve the equation for *c* and the function *immse* to calculate the mean square error between the simulated and experimental data (the value was multiplied by 100,000 to avoid numerical issue with small numbers). The fitting was performed by the *patternsearch* function of the Global Optimization toolbox. The initial guess was a diffusion constant of 10^−3^ μm^2^ s^−1^, converted into units of pixels, the length unit on which the solver was run. Our results were not sensitive to this choice. Applying this procedure, we obtain a spatially varying effective diffusion constant for each cell. The swarmplots in Fig. 3f show the median of the profile for dividing and non-dividing cells while those in Supplementary Fig. 5b also include the mutant strains. These plots were created using the web application https://www.estimationstats.com as described in Ho et al.^45^. Each cell has a different length and so to combine the data from all cells (for a given mutant/dividing state), we re-express the effective diffusion constant in terms of the relative, rather than absolute, position in the cell, using interpolation (via *interp1*) to obtain this profile at the same 51 relative positions (0, 0.02, …, 1) for each cell. We then combine the spatial profiles from all cells (Fig. 3g and Supplementary Figs. 5d and 6c). Shown is the median with its bootstrapped 95% confidence interval, found using the Matlab function *bootci*. Note that non-overlapping confidence intervals indicates significance.

### Western blotting

Cultures were grown as described in sample preparation for live microscopy. Aliquots of 1 ml were centrifuged for 3 min at 10,000 × *g* and resuspended in 30 µl of Laemmli sample buffer and denatured for 15 min to lyse the cells. Samples were run on a 15% SDS-PAGE gel (30 mA, 30 min), blotted on Sequi-Blot PVDF membrane (Bio-Rad #1620182) and blocked with 8% Marvel dried skimmed milk in Tris-buffered saline buffer with Tween 20 (TBST buffer) overnight at room temperature. Blots were probed with primary rabbit anti-Pal (1:1000) and anti-TolB (1:500) antibodies in 4% milk in TBST buffer for 1 h at room temperature. Membranes were then washed with TBST buffer (5 × 1 min) and probed with secondary goat anti-rabbit antibodies conjugated with peroxidase (1:1000, Sigma #A6154). Blots were washed as described above and detection was carried out using Amersham ECL Western Blotting Select Detection Reagent (GE Lifesciences #RPN2235), according to the manufacturer’s instructions in GBOX-CHEMI-XRQ. Images were recorded using GeneSys software.

### Protein expression and purification

The C-terminal domain of *Pseudomonas aeruginosa* TolA (TolA^224–347^) and its derivatives were expressed as fusion proteins with an N-terminal, His-tagged Im9 in *E. coli* BL21 (DE3) cells after induction with 1 mM IPTG 37 °C. After 4 h growth, cells were harvested by centrifugation (4000 × *g*, 4 °C) and resuspended in binding buffer (50 mM Tris, 1 mM MgCl_2_, 2 mM imidazole, 1 mM PMSF (Sigma), 300 mM NaCl, pH 7.5, with protease inhibitor cocktail (Roche cOmplete Easypack tablets)). Cells were lysed by sonication on ice (Misonix Sonicator 4000). Cell debris was removed by centrifugation (Beckman JA-25.50, 48,000 × *g*, 4 °C, 30 min), and the supernatant loaded onto a freshly charged 5 ml HisTrap FF Ni-affinity column (GE Healthcare) at 1 ml min^−1^ at 4 °C (50 mM Tris buffer, 50 mM NaCl, 2 mM imidazole, pH 7.5) and eluted using a linear gradient of imidazole (2−250 mM). Fractions containing Im9-TolA^224–347^ were pooled and dialysed overnight against 50 mM Tris, 100 mM NaCl, 5 mM CaCl_2_ pH 7.5 at 4 °C. The sample was concentrated to ~5 mg ml^−1^ in a 10 kDa spin concentrator (Vivaspin) and the Im9-TolA^224–347^ fusion cleaved overnight at 4 °C with 150 units of Novagen FXa at a ratio of 2 units per mg of fusion protein. Cleaved TolA^224–347^ was purified by Ni-affinity FF His-Trap as above. PMSF (1 mM) was added to pooled fractions containing TolA^224–347^ and the concentrate dialysed against 50 mM Tris, 100 mM NaCl, pH 7.5 at 4 °C before further purification on an S75 Superdex 26/60 column, equilibrated in 50 mM Tris, 100 mM NaCl, pH 7.5 at 4 °C. The purified protein was snap-frozen in liquid nitrogen and stored at −80 °C. Uniformly ^15^N-labelled or ^15^N- and ^13^C-labelled Im9-TolA^224–347^ was prepared with M9 growth media (0.0477 M Na_2_HPO_4_, 0.022 M KH_2_PO_4_, 8.6 mM NaCl, 4 μM ZnSO_4_, 1 μM MnCl_2_, 0.7 μM H_3_BO_3_, 0.7 μM CuSO_4_, 2 μM FeCl_3_, 2 mM MgSO_4_, 0.1 mM CaCl_2_, 0.3% (w/v) glucose (^13^C glucose from Sigma), 0.1% (w/v) ammonium chloride (^15^N NH_4_Cl from Sigma)).

Unlabelled *E. coli* or *P. aeruginosa* TolB^22–34^ (the N-terminus of secreted TolB) and mutant variants were used as synthetic peptides with amidated C-termini (thinkpeptides® UK (ProImmune)). TolB^22–34^ peptides were typically only soluble up to ~3 mM in the buffered solutions. For fluorescence anisotropy experiments, *P. aeruginosa* TolB^22–34^ was labelled with fluorescein isothiocyanate through a C-terminal lysine residue incorporated into the peptide sequence. The C-terminal end of TolB^22–34^ plays no role in TolA binding.

^13^C/^15^N-labelled *P. aeruginosa* TolB^22–34^ was generated by expressing the TolB sequence from the pMMHb plasmid as a fusion with the TrpLE leader sequence which contains an intervening methionine residue for cyanogen bromide cleavage and release of the peptide^46,47^. Protein expression in M9 media was induced with 1 mM IPTG and cultures grown overnight at 37 °C to a final OD_600_ of ~1.4. Protein was purified from inclusion bodies solubilized in 6 M Gn.HCl, 25 mM Tris pH 8.0, 5 mM imidazole. Following centrifugation, supernatant was loaded onto a 5 ml Ni-affinity column (GE HisTrap) equilibrated in 6 M guanidine, 25 mM Tris pH 8.0, 5 mM imidazole. Protein was eluted from the column stepwise (25–500 mM imidazole in 6 M guanidine, 25 mM Tris pH 8.0). Column fractions were dialysed against 4 l MilliQ water over 2 days to precipitate protein which was then pelleted. The TolB^22–34^ peptide was chemically cleaved by dissolving the pellet into 5 ml 70% v/v formic acid and 0.5 g CNBr added, kept under nitrogen and the reaction allowed to continue for 2.5 h. Cleavage was quenched by a tenfold dilution with water followed by flash freezing and lyophilization. Recombinant peptide was purified by reverse phase HPLC. TolB^22–34^ was eluted on a linear gradient of 0−95% v/v acetonitrile with 0.1% v/v TFA using a Dionex 218TP C18 VYDAC column attached to an AKTA Purifier. Cleavage efficiency was routinely ~80−90%. Fractions corresponding to peptide were confirmed by mass spectrometry.

### Protein concentration

Protein concentrations were determined by UV absorbance at 280 nm using the method of Gill and von Hippel^48^.

### Nuclear magnetic resonance spectroscopy

NMR experiments were carried out using spectrometers operating at ^1^H frequencies ranging from 500 to 950 MHz. The spectrometers were equipped with Oxford Instruments magnets and home-built triple-resonance pulsed-field gradient probes (600, 750 and 950 MHz) or with Bruker Avance consoles and TCI CryoProbes (500 and 600 MHz). NMR data were acquired using either GE/Omega software using pulse sequences written in-house, or Topspin software and pulse sequences in the Topspin libraries from Bruker Biospin. All data processing was conducted using NMRPipe^49^. Processed spectra were visualized and assigned using the CCPN software suite^50^. All NMR data were collected at 20 °C.

Resonance assignments for TolA^224–347^ bound to TolB^22–34^ were obtained using standard 2D and 3D double and triple resonance data including ^15^N-HSQC, ^13^C-HSQC, ^15^N-edited TOCSY-HSQC, HNCO, HNCACO, HNCA, CBCANH, CBCA(CO)NH, HBHA(CBCACO)NH, H(CCCO)NH, (H)CC(CO)NH and HCCH-TOCSY. The NMR samples contained 0.6 mM 15N or 13C/15N-labelled TolA^224–347^ and 3 mM unlabelled TolB^22–34^. Resonance assignments for the TolB^22–34^ peptide in complex with TolA^224–347^ were obtained using 2D versions of some of the experiments listed above using a sample containing 0.2 mM ^13^C/^15^N-labelled TolB^22–34^ and 1.1 mM unlabelled TolA^224–347^. Details of the assignments are included in BMRB deposition 27397.

Residual dipolar couplings (RDCs) for ^15^N-TolA^224–347^ bound to TolB^22–34^ were measured using a sample aligned in 5% w/v C_12_E_6_ PEG/*n*-hexanol and the IPAP NMR experiment^51,52^. Values for the axial and rhombic components of the alignment tensor were estimated initially from the shape of the distribution of RDC values and then refined by fitting using in-house software and the previously determined X-ray structure of free TolA (PDB: 1LR0) and the RDC values for regions of secondary structure not involved in peptide binding; *D*_a_/*R* values of 13.7/0.01 were used for the structure calculations.

### Structure determination

The structure of TolA^224–347^ in complex with TolB^22–34^ was determined using ARIA (version 2.3), interfaced to CNS (version 1.2)^53–57^. NOE distance restraints were set as ambiguous restraints in early calculations, peak ambiguity was decreased during eight iterations before a final water refinement calculation. Distance restraints were derived from NOE peak intensities in ^15^N-edited NOESY-HSQC, ^15^N-edited HSQC-NOESY-HSQC ^13^C-edited NOESY-HSQC (collected in 95%H_2_O/5%D_2_O and in 100% D_2_O) and ^13^C,^15^N isotope-filtered ^13^C-separated NOESY experiments; analysis of these data sets allowed protein−protein, protein−peptide and peptide−peptide NOEs to be distinguished. Backbone *ϕ* and *ψ* torsion angles for TolA^224–347^ and TolB^22–34^ were estimated from Cα, Cβ, C′, N and Hα chemical shifts using the program DANGLE^58^. Hydrogen bond restraints were based on slowly exchanging amides identified in ^15^N-HSQC spectra collected in D_2_O and observed NOEs characteristic of regular secondary structure. A square well potential and a force constant of 0.5 were used for the RDC restraints (SANI terms) with experimental error for the RDCs set to 2 Hz. In the final ARIA iteration, 600 structures were calculated; the 20 lowest energy structures were selected for a final round of water refinement. The family of structures was then validated using PROCHECK-NMR.

### Isothermal titration calorimetry

All ITC experiments were performed using the C-terminal domain of *E. coli* TolA (TolA^224–347^). TolA was dialysed into 50 mM Hepes buffer, 50 mM NaCl at pH 7.5 overnight at 4 °C and precipitates removed by centrifugation (10,000 × *g*, 10 min). The concentration was adjusted to 150 μM. TolA was loaded into the cell and synthetic TolB peptide (residues 22−33; 2 mM) loaded into the syringe of an iTC200 microcalorimeter (Microcal/GE Healthcare). Titration consisted of 20 injections (1 × 0.4 μl, 19 × 2 μl) measured at 25 °C with an interval of 150 s between injections, and a stirring speed of 1000 rpm. Heats of dilutions were measured by injecting syringe samples into buffer under identical titration conditions and subtracted from each data set. Data were analysed using Origin 7.0 software, and fitted to a single site binding model.

### Fluorescence anisotropy

Fluorescence anisotropy experiments were used to observe the change in overall anisotropy of *P. aeruginosa* TolB^22–34^ fluorescein isothiocyanate (FITC) in complex with the C-terminal domain of TolA. A titration curve was recorded using 40 μl fractions in a 96-well, black absorbent, 100 μl plate, *n* = 3. Data analysis was performed in SigmaPlot 12.0, using a non-linear regression dynamic curve fit using the quadratic equation:

$$f = ({\mathrm{{A}}}_{\mathrm{{T}}} + {\mathrm{{B}}}_{\mathrm{{T}}} + k \pm \surd (({\mathrm{{A}}}_{\mathrm{{T}}} + {\mathrm{{B}}}_{\mathrm{{T}}} + k)^2 - 4{\mathrm{{A}}}_{\mathrm{{T}}}{\mathrm{{B}}}_{\mathrm{{T}}})){\mathrm{/}}2,$$

where *f* = concentration of TolA-TolB, A_T_ = Total concentration of TolA, B_T_ = Total concentration of TolB, and *k* = *K*_d_.

Experiments were carried out using either a CLARIOstar plate reader or a Fluoromax- 4 spectrofluorimeter (Horiba JobinYvon). Titrations were carried out between 0 and 1.2 mM TolA. The fluorophore target was a fluorescein isothiocyanate-labelled TolB^22–34^ peptide (Proimmune). FITC excitation wavelength used was 495 nm with an emission wavelength at 519 nm. All experiments were conducted in 50 mM Tris 100 mM NaCl pH 7.0 and used light-blocking tubes to minimize quenching.

Stopped-flow anisotropy was used to determine on- and off-rates of the TolA-TolB complex. Experiments were conducted on an Applied Photophysics SX20 instrument set up for 1:1 single mixing and thermostated using a circulating water bath at 25 °C. All stopped-flow experiments were carried out in 50 mM Tris pH 7.0, 100 mM NaCl and under pseudo-first-order conditions. To observe anisotropy of FITC fluorescence, the apparatus was set up using an SX/FP polarization accessory. FITC was excited at 470 nm and emission was monitored above 515 nm using cut-off filters at both emission channels in the T-mode. Monochromator entrance and exit slits were set to 2 mm. A total of 4000–10,000 data points were collected for each reaction. At least four anisotropy traces were collected for each reaction and averaged for each time-dependence fit. All anisotropy traces were fit to a single exponential rate equation. Each reaction concentration was collected in triplicate and validated using recombinant protein from different protein purifications. SigmaPlot was used for linear regression analysis to fit the dependence of *k*_obs_ on the varied concentration of TolA. Titrations were performed at increasing concentration of protein relative to a constant concentration of peptide at 1 μM. Experiments were typically performed with 4–10-s data collection periods.

### Assessing the stability of the *E. coli* OM

The stability of the *E. coli* outer membrane was assessed for *tol* phenotype and impaired growth in the presence of SDS, for engineered strains and plasmid-transformed strains. Overnight cultures were used to inoculate 5 ml LB cultures with appropriate antibiotic and subsequently brought to log phase (OD_600_ ~ 0.4). Aliquots of these cultures were then spotted at regular intervals on SDS-containing plates (0.4 and 2%) in serial dilutions of spotted cell densities, from 0.02 to 0.000002 using the initial OD_600_ measurement. Plates were then incubated at 37 °C overnight. Images were recorded in GBOX-CHEMI-XRQ, using GeneSys software. Cultures unable to grow in the presence of 2% SDS are deemed to have destabilized outer membranes.

### Growth comparison of mCherry/PAmCherry-Pal strains

Overnight cultures grown in M9/glucose with appropriate antibiotics were diluted in fresh medium to OD 0.04 and grown at 37 °C. Culture density was measured every hour using Biochrom WPA CO8000 Cell Density Meter which was then plotted as a function of time.

### Steered molecular dynamics simulations of the TolB-Pal complex

The structure of the *E. coli* TolB-Pal complex was obtained from the Protein Data Bank (PDB: 2W8B)^18^. The disordered N-terminus of Pal, not present in the crystal structure, was modelled using Modeller 9.19^59^ and attached to the tripalmitoyl-*S*-glyceryl-cysteine residue as previously described^60^. The tripalmitoyl-*S*-glyceryl-cysteine residue was then inserted into the inner leaflet of an *E. coli* outer membrane model^61^. Mg^2+^ ions were added to facilitate crosslinking between lipopolysaccharide head groups in the membrane. The system was then solvated with the SPC water molecules^62^ and neutralised with 0.15 M NaCl. A short 10 ns equilibration simulation was performed whereby all the heavy atoms of the proteins were positionally restrained using a force constant of 1000 kJ mol^−1^. The temperature was maintained at 310 K using a velocity rescale thermostat^63^, while the pressure was kept at 1 atm using a semi-isotropic pressure coupling to a Berendsen barostat^64^. Long-range electrostatic interactions were calculated using the particle mesh Ewald method^65^, with the short-range electrostatic and van der Walls cut-offs set to 1.4 nm. A time step of 2 fs was used as all bonds were constrained using the LINCS algorithm^66^.

After the equilibration simulation, the positional restraints on the protein were removed and a 100 ns equilibrium simulation was performed to allow the N-terminal linker of Pal to contract, resulting in interactions between Pal and the outer membrane. All simulation parameters were maintained except the pressure coupling, which was changed to the Parrinello−Rahman barostat^67^. The final snapshot of this simulation was used to denote the bound configuration of the TolA binding site in the TolB-Pal complex. To generate the configuration in which the TolA binding site of TolB was disordered, a steered MD simulation was performed whereby a harmonic spring with a force constant of 1000 kJ mol^−1^ nm^−2^ was attached to residue Glu22 (the N-terminus) and pulled along the *z*-axis (perpendicular to the plane of the membrane) at a constant velocity of 0.5 nm ns^−1^. This resulted in the detachment of the TolA binding site from the surface of the TolB β-propeller domain.

To estimate the force required to dissociate the TolB-Pal complex in the two configurations, further steered MD simulations were performed. A harmonic spring with a force constant of 1000 kJ mol^−1^ nm^−2^ was attached to the centre of mass of TolB and pulled along the *z*-axis away from Pal at a constant velocity of 0.05 nm ns^−1^. All the heavy atoms in Pal were positionally restrained using a force constant of 1000 kJ mol^−1^. Five independent steered MD simulations with different initial velocity distributions were conducted for each configuration, and average force and standard deviation along the pulling coordinates were calculated. The same protocol was repeated with a pulling speed of 0.5 nm ns^−1^, which yielded a similar trend in forces required to dissociate TolB from Pal in the two TolA box configurations. All simulations were performed using the GROMACS 2018 code^68^ with the GROMOS 54A7 forcefield^69^ and visualized in VMD^70,71^.

### Reporting summary

Further information on research design is available in the Nature Research Reporting Summary linked to this article.

## Supplementary information

Supplementary information

Reporting Summary

## Data Availability

The data supporting the findings of the study are available in the article and its Supporting Information or archived in the PDB or available upon request from the corresponding author. The source data underlying Figs. 1–3d, 4a, 4e and Supplementary Figs. 2b, 3 and 4 are provided as a Source Data file. The source data for Fig. 3f, g and Supplemental Figs. 5 and 6 are available on the GitHub repository (https://github.com/smury/SpatialFRAP) together with the Matlab scripts. The coordinates of the family of 20 NMR structures of *P. aeruginosa* TolA-TolB complex have been deposited in the Protein Data Bank under accession number 6S3W. Resonance assignments for TolA-TolB have been deposited in the BioMagResBank (BMRB) under accession number 27397.

## Source data

Source Data

## Code availability

The custom Python scripts used for selecting and processing single-particle tracking data (Fig. 1b, c) are available upon request from the corresponding author. Matlab scripts for the SpatialFRAP analysis (Fig. 3f, g and Supplemental Figs. 5 and 6) are available on the GitHub repository (https://github.com/smury/SpatialFRAP).
