# Supplementary material for: The lipoprotein Pal stabilises the bacterial outer membrane during constriction by a mobilisation-and-capture mechanism
Source: Nat Commun. 2020 Mar 11;11:1305. doi: 10.1038/s41467-020-15083-5 (PMC7066135; doi:10.1038/s41467-020-15083-5)
Supplement: Supplementary file 1 — Supplementary information [file 41467_2020_15083_MOESM1_ESM.pdf]

SUPPLEMENTARY INFORMATION

The lipoprotein Pal stabilises the bacterial outer membrane during constriction by a mobilisation-and-capture mechanism

Joanna Szczepaniak[1,‡], Peter Holmes[1,¶,‡], Karthik Rajasekar[1,#,‡], Renata Kaminska[1], Firdaus Samsudin[2], Patrick George Inns[1], Patrice Rassam[1,†], Syma Khalid[2], Seán M. Murray[3], Christina Redfield[1] & Colin Kleanthous[1*]

[1]Department of Biochemistry, University of Oxford, Oxford, OX1 3QU, UK
[2]Department of Chemistry, University of Southampton, University Road, Southampton, SO17 1BJ, UK
[3]Max Planck Institute for Terrestrial Microbiology and LOEWE Centre for Synthetic Microbiology (SYNMIKRO), Karl-von-Frisch Strasse 16, 35043 Marburg, Germany
[‡] These authors contributed equally

¶Present address: Department of Biochemistry, University of Alberta, Edmonton, Alberta, T6G 2H7, Canada
#Present address: Evotec SE, 112-114 Innovation Drive, Milton Park, Abingdon OX14 4RZ, UK
†Present address: Laboratoire de Bioimagerie et Pathologie, UMR 7021, CNRS, Université de Strasbourg, Faculté de pharmacie, 74 Route du Rhin, 67401 Illkirch, France

[*]Address for correspondence:
Prof Colin Kleanthous, Department of Biochemistry, South Parks Road, University of Oxford, Oxford OX1 3QU, U.K. Tel: +44-1865-613370. Email: colin.kleanthous@bioch.ox.ac.uk

Supplementary Figure 1

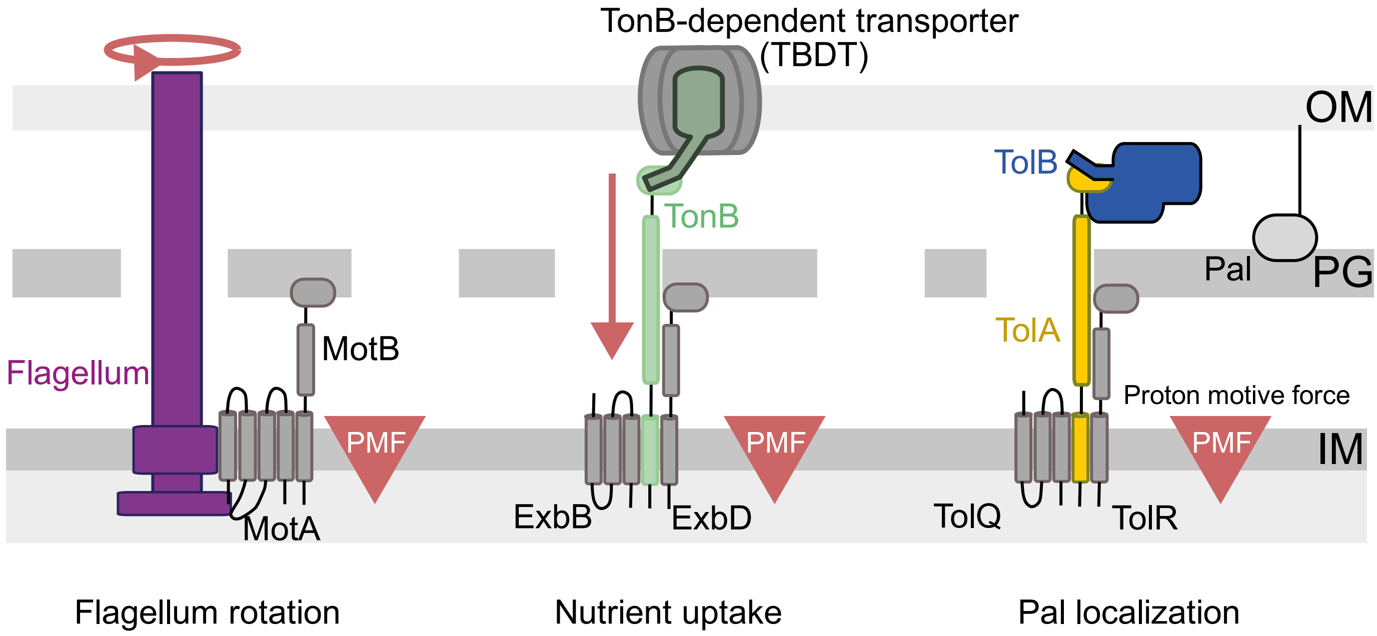

Supplementary Figure 1. Schematic illustrating the comparative PMF-harvesting components of Tol with Ton and the flagellum all of which span the bacterial cell envelope. All three systems have related PMF-harvesting proteins in the inner membrane – TolQ and TolR in Tol, ExbB and ExbD in Ton and MotA and MotB in the flagellum – and conserved transmembrane residues couple the PMF to mechanical motion (not depicted). In the case of the bacterial flagellum, multiple MotA/MotBs are recruited to drive rotation of the flagellum[72]. In the case of Ton, TonB is activated by the PMF through ExbB and ExbD to displace the plug domains of ligand-bound TBDTs, which typically transport iron siderophore complexes or vitamin B_{12} into the periplasm[28]. Contact with the TBDT is through a TonB box sequence at the N-terminus of the TBDT that interacts with the C-terminal domain of TonB close to the outer membrane. In the case of Tol, TolQ and TolR cause PMF-mediated conformational changes to TolA, which in turn interacts with the N-terminus of TolB[18].

Supplementary Figure 2

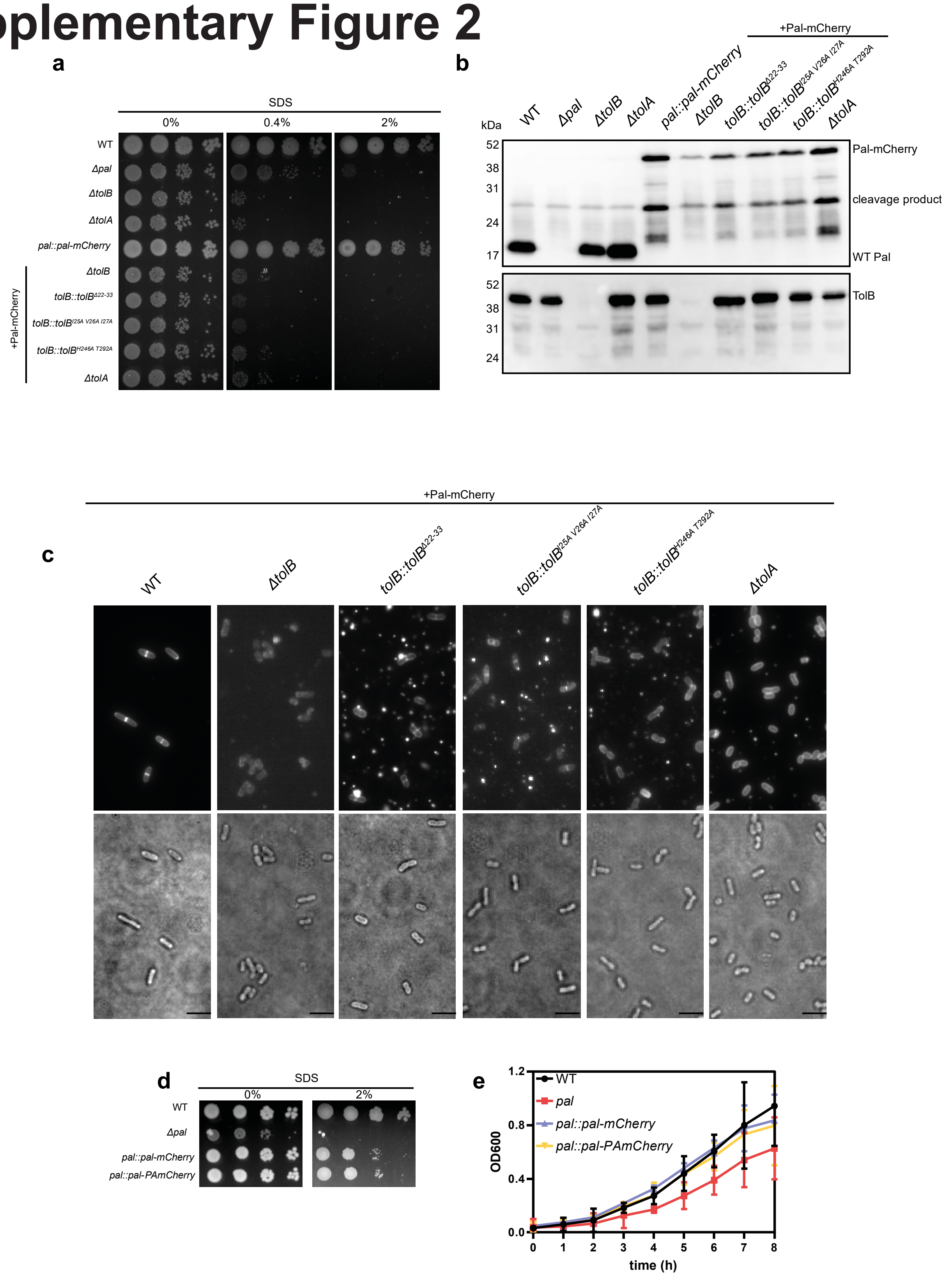

Supplementary Figure 2. *E. coli* phenotypes of engineered strains and expression levels of Pal. ***a,*** Representative images from OM stability assays. Cultures were grown at 37°C, serially diluted in fresh LB medium, pipetted onto LB plates containing 0, 0.4 and 2% SDS and grown overnight at 37°C. Experiments were repeated three times (representative image shown from one experiment). Only wild-type (WT) and complemented Pal-mCherry strains show growth on 2% SDS. ***b,*** Representative Western blots probed with anti-Pal or anti-TolB antibodies. mCherry protein is cleaved at the chromatophore which after sample denaturation results in two bands on the Western blot; full-length Pal-mCherry and Pal linked to the cleaved C-terminus of mCherry. This cleavage does not impact the fluorescence in native conditions[73]. All *tolB* mutants show lower protein levels of Pal. ***c,*** Representative images of TIRFM fields of view of the strains used in this work. Outer membrane vesicles (OMVs) are evident for tol mutants where they appear as fluorescent vesicles amongst cells. OMV production has long been associated with mutations in the Tol assembly[74]. Contrast/brightness levels are set individually for each image. Scale bar, 5 µm. ***d,*** Representative images from OM stability assays for *pal::pal-PAmCherry* strain. Experiments were repeated three times (representative image shown from one experiment). ***e,*** Growth comparison of mCherry/PAmCherry tagged strains. The curves are average from three experiments, with standard deviation

Supplementary Figure 3

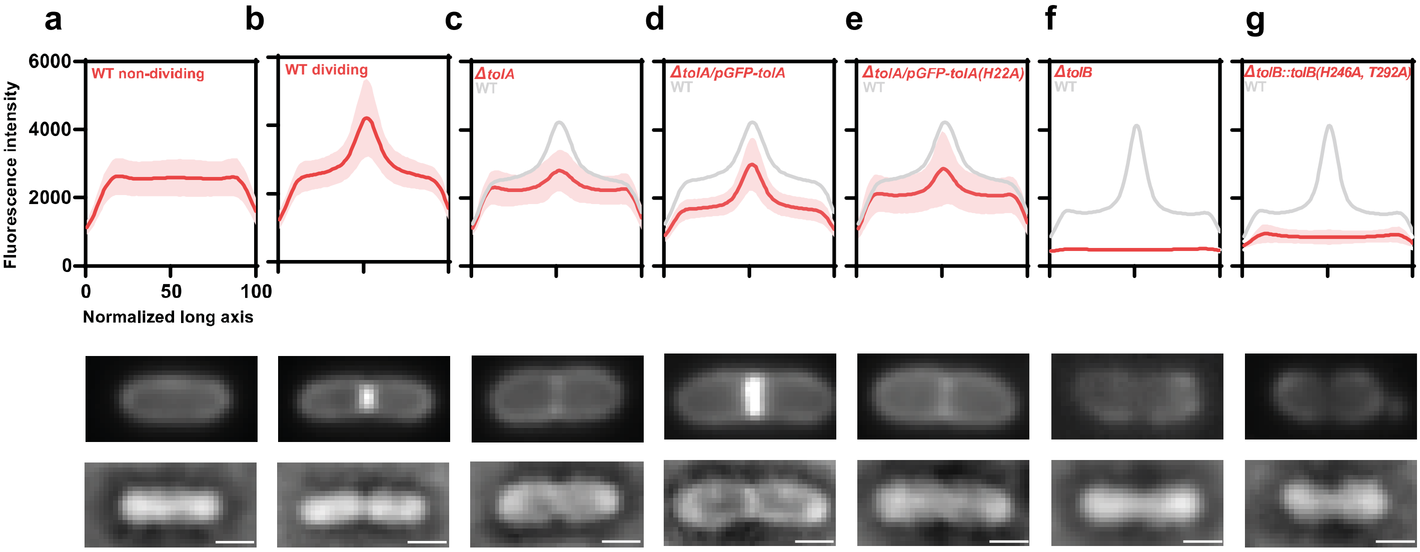

Supplementary Figure 3. Pal-mCherry distribution profiles in wild-type and tol mutant *E. coli* cells. Average distribution of Pal-mCherry along the normalized x-axis of the cells. In each case, curves are averages from 30 cells, with shaded areas representing the standard deviation. Representative TIRFM and brightfield images of dividing cells are shown beneath each set of data. Contrast/brightness levels are set individually for each image. Scale bar throughout, 1 µm. ***a,*** Wild-type non-dividing cells. The peripheral distribution of the Pal-mCherry fluorescence in non-dividing cells is consistent with the periplasmic location of Pal. **b,** Wild-type dividing cells. ***c,*** *tolA* deletion strain. ***d,*** *tolA* deletion strain expressing GFP-TolA from an IPTG-inducible plasmid. ***e,*** *tolA* strain expressing GFP-TolA with H22A mutation that decouples TolA from the PMF. **f,** *tolB* deletion strain. ***g,*** *tolB* strain expressing chromosomal TolB with mutations in H246A and T292A that abolish TolB binding to Pal.

Supplementary Figure 4

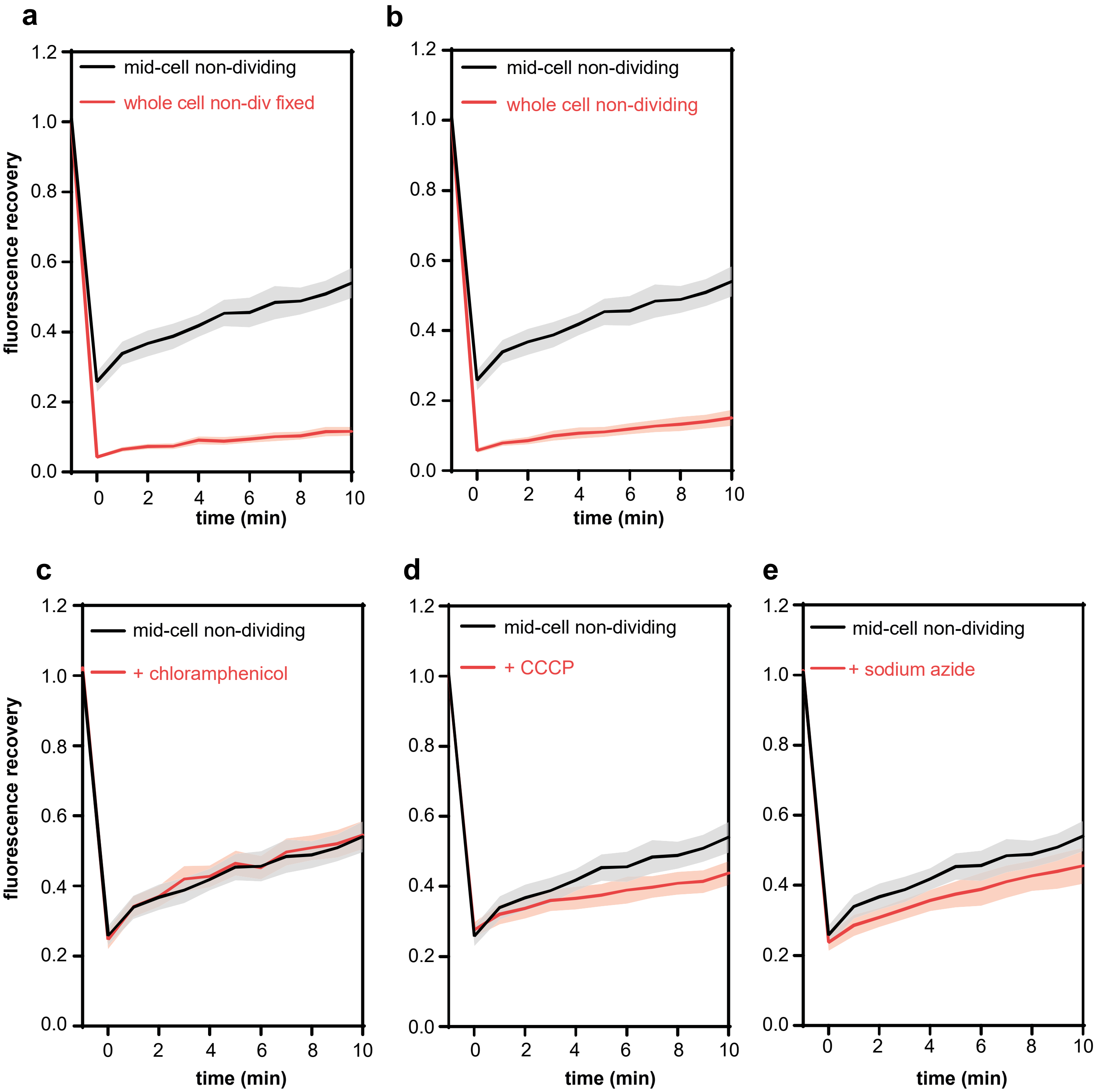

Supplementary Figure 4. Biogenesis of Pal and mCherry photoswitching have a small effect on fluorescence recovery in FRAP experiments. Due to the long timescale of the FRAP experiments the observed fluorescence recovery in our experiments may be influenced by Pal synthesis, its insertion in the OM and/or fluorophore switching back to its fluorescent state. ***a,*** To assess the impact of mCherry photoswitching, whole formaldehyde-fixed non-dividing cells were bleached, and their fluorescence recovery was normalized to a non-bleached cell acquired in the same field of view. As a control, live non-dividing cells were also bleached in the rectangular region of interest set at the mid-cell. ***b,*** Live non-dividing cells were bleached as described above. The levels of recovery suggest that Pal-mCherry biogenesis and Pal insertion in the OM make a relatively minor contribution to FRAP data. ***c,*** To address the issue of Pal biogenesis on recovery curves, non-dividing cells were treated with 30 μg/μl of chloramphenicol and bleached at mid-cell. The recovery was identical to that of untreated cells. ***d* and *e,*** Addition of CCCP (0.1 mM) or sodium azide (50 μg/μl) had a measurable effect on FRAP recovery curves suggesting biogenesis/secretion impact fluorescence recovery in our FRAP experiments data but that their contributions are relatively minor. These factors account for a quarter of the observed recovery in live cells, which reiterates that the bulk of Pal-mCherry fluorescence recovery in FRAP experiments is due to slow Pal mobility in the OM of *E. coli.* In all experiments, recovery curves are an average from 30 cells, with shaded area representing 95% CI, compared to recovery curve of bleached mid-cell of non-dividing cells.

Supplementary Figure 5

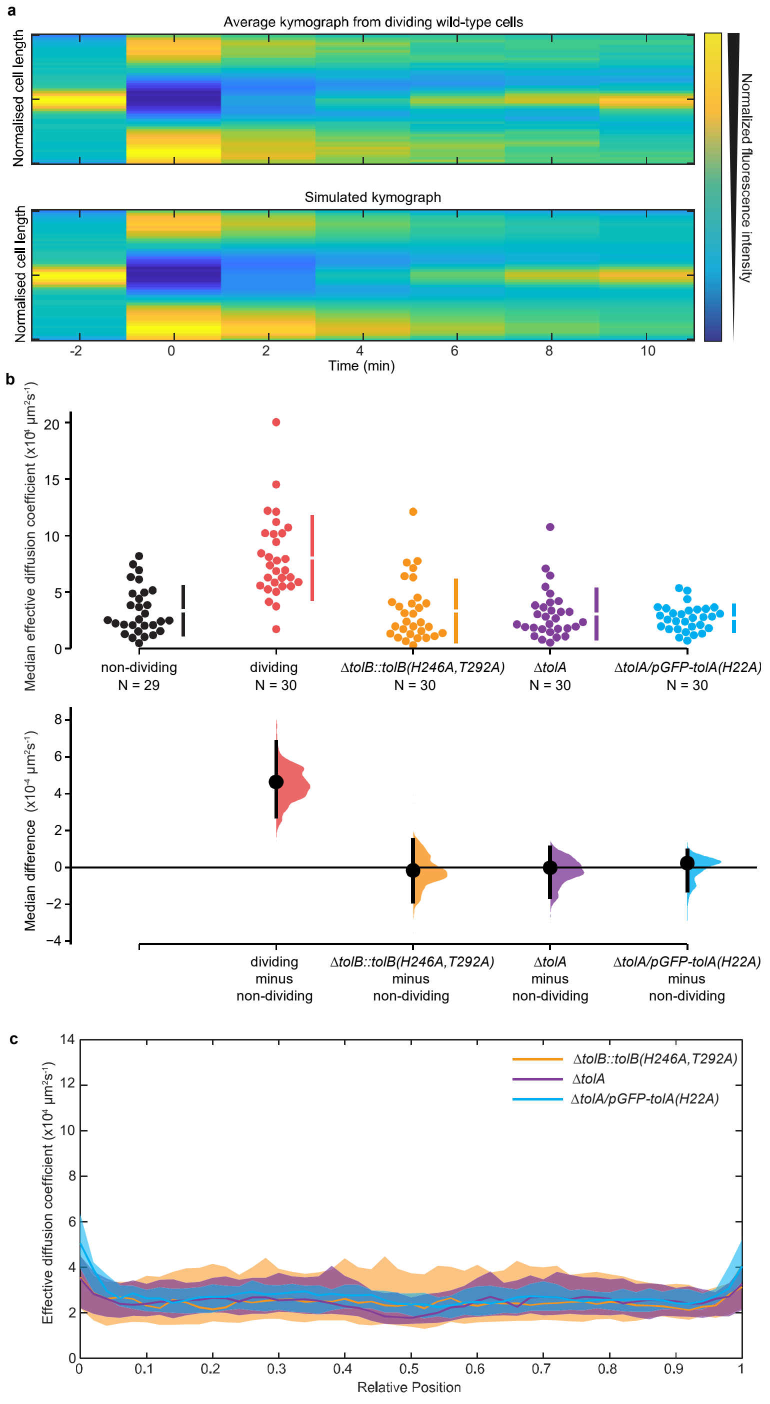

Supplementary Figure 5. SpatialFRAP: a method for obtaining a spatially varying effective diffusion coefficient (D_{eff}). *a,* Top, average kymograph from 30 dividing wild-type cells (as analysed in **Figures 2 and 3**). Colour indicates normalised fluorescence. All cells were normalized to the same cell length before averaging. Bottom, simulated kymograph obtained by fitting simulated data to the data above. The prebleach frame (-2 min) was used to specify the shape of D_{eff} up to a scaling factor. This scaling factor was then obtained by the numerical solution of the equation given in the methods to the experimental data, taking the prebleach frame (0 min) as the initial condition. See **Methods** for further details. The median D_{eff} obtained above is ~8.7 x 10^{-4} $\mu m^2 s^{-1}$, which is a rough estimate because of the varying cell lengths. The data in **Figure 3f, g** and below were obtained by performing the same procedure on individual cells. ***b,*** The effective diffusion coefficient of Pal-mCherry in wild-type dividing and non-dividing cells (from **Figure 3f**) along with *tolB* and *tolA* mutant strains. Shown is a Cumming estimation plot. The raw data are plotted on the upper axes with vertical bars indicating standard deviations. On the lower axes, the median differences with non-dividing cells are plotted as bootstrap sampling distributions (5000 samples), with each mean difference depicted as a dot and 95% confidence intervals indicated by the ends of the vertical bars. The confidence interval is bias-corrected and accelerated. None of the mutated strains show a significant difference against non-dividing cells ***c,*** D_{eff} of Pal-mCherry as a function of cellular location in dividing *ΔtolB::tolB(H246A T292A), ΔtolA* and *ΔtolA/pGFP-tolA(H22A)* cells obtained from FRAP data as in **Figure 3g**. Unlike the WT condition (**Figure 3g**), the spatial profile of D_{eff} within cells is similar to that in non-dividing cells.

Supplementary Figure 6

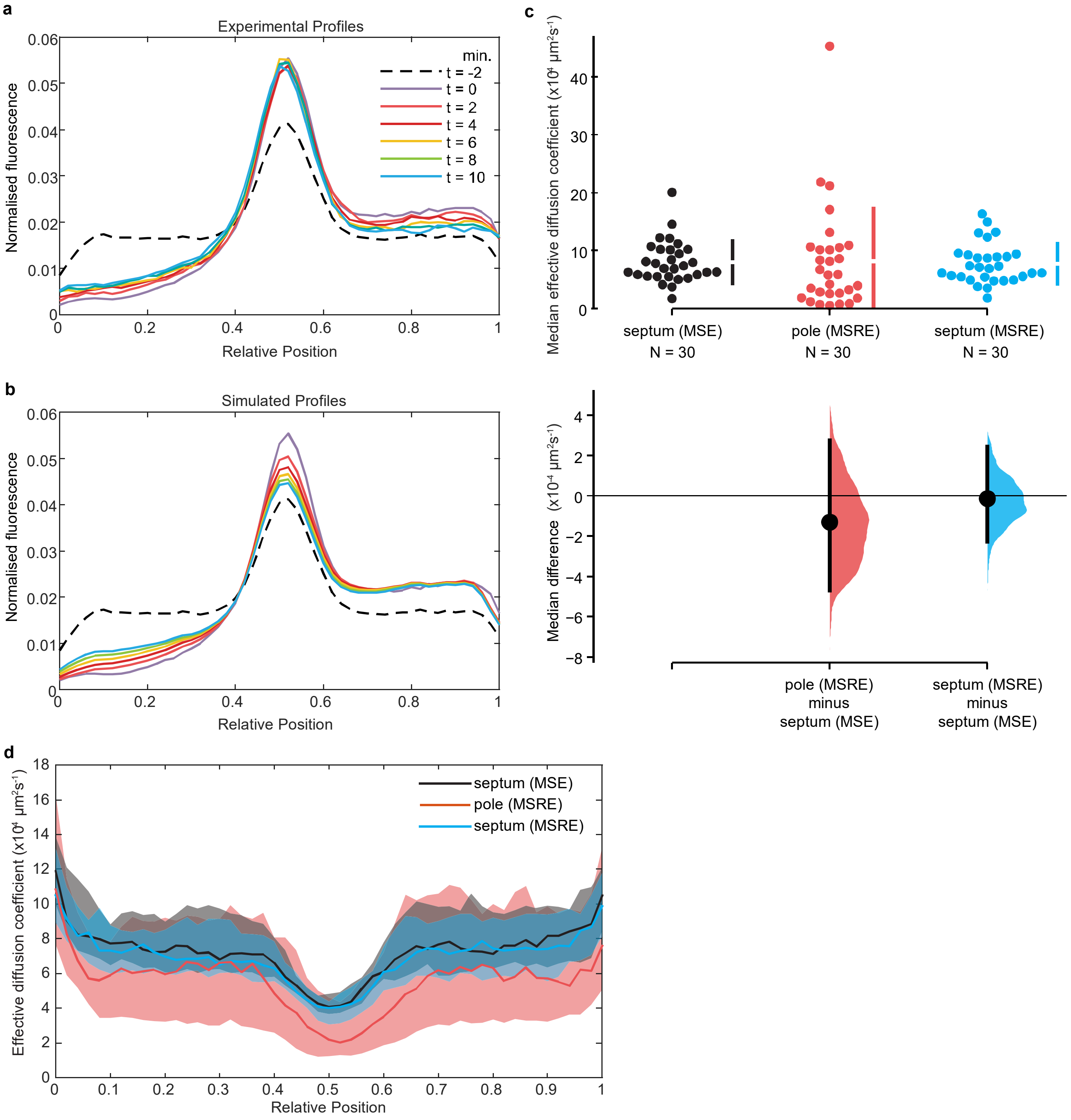

Supplementary Figure 6. Polar FRAP analysis of Pal-mCherry expressing *E. coli* cells. As a control of the SpatialFRAP analysis method, dividing cells were bleached not at the septum, as in **Figure 3**, but at one of their poles. ***a,*** Average recovery profiles of Pal-mCherry from 30 cells, scaled to cell length of 1. The pole at relative position 0 was bleached immediately before the image taken at t=0 min. Note the slow recovery of the bleached pole. ***b,*** Simulated recovery profiles obtained by fitting to the average profiles in ***a.*** The fits in **Figure 3f, g** and **Supplementary figure 5** used the mean square (absolute) error (MSE) between the experimental and simulated profiles as the cost function. The MSE weights the percentage error towards regions with high signal as is generally appropriate for noisy data. However, here we use the mean square relative error (MSRE) as otherwise the fit is biased towards the high signal at the septum and against the consistently weak signal at the bleached pole, which is where the temporal information is primarily located. With the MSRE, a deviation of 10% is treated the same, whether it occurs at the septum or at the pole. ***c,*** The median effective diffusion coefficient of Pal-mCherry in individual wild-type dividing cells obtained by bleaching either the septum (same data as in **Figure 3f**) or a pole. Shown is a Cumming estimation plot as in **Supplementary Figure 5b**. No significant difference was found between cells bleached at the pole and at the septum. The fitting method (MSE or MSRE) had no effect on the result for septum-bleached cells. ***d,*** D_{eff} of Pal-mCherry as a function of relative cellular location in dividing cells. Shown is the median across all cells. Shading indicates the 95% confidence interval calculated using 1000 bootstrapped samples.

Supplementary Figure 7

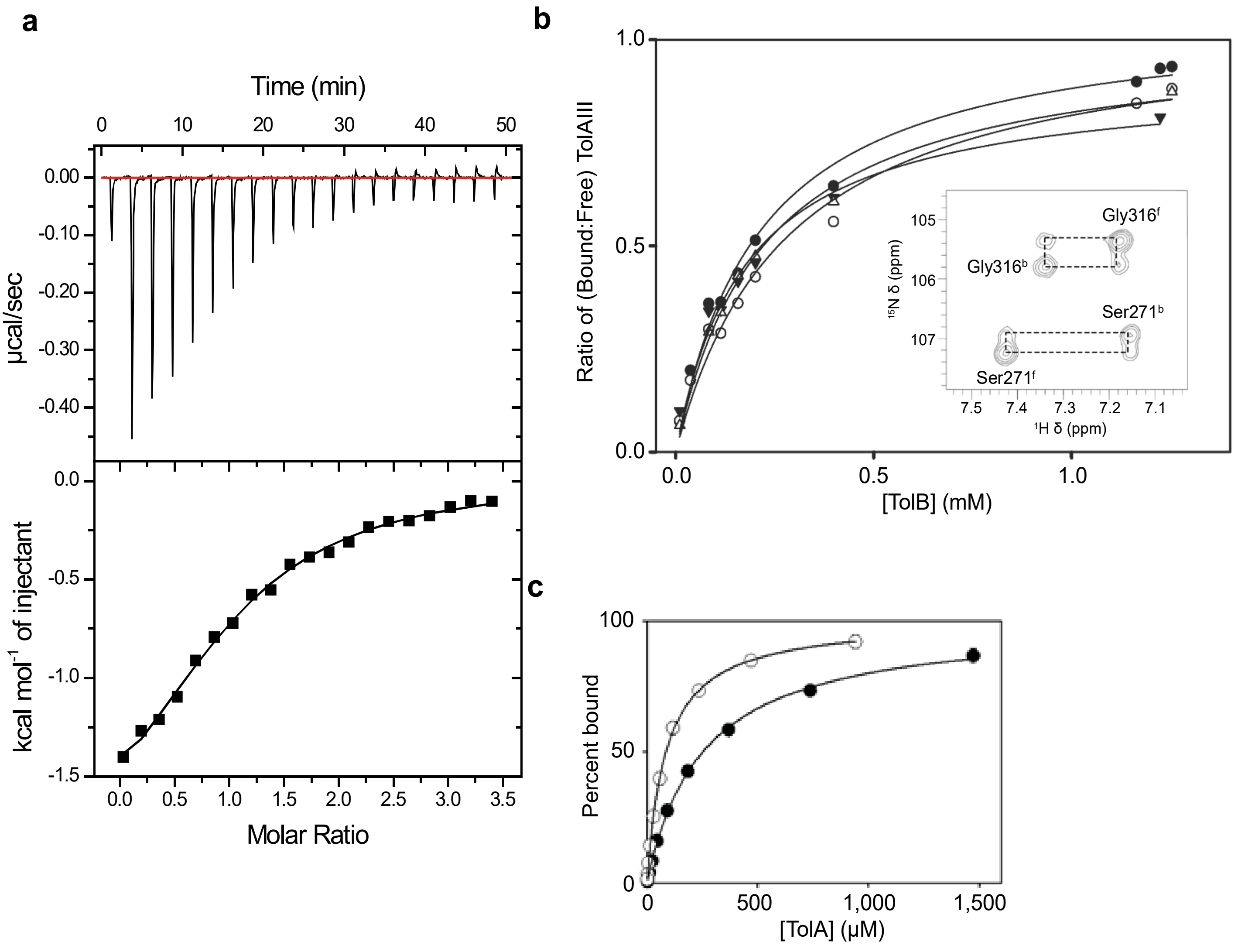

Supplementary Figure 7. The N-terminus of TolB binds weakly to the C-terminal domain of TolA. ***a,*** ITC data of *E. coli* TolA C-terminal domain into which was injected a 12-residue TolB peptide (TolB$^{22-33}$). Data were fitted using the manufacturer's software assuming a 1:1 binding model, validated by the resulting structure for the complex. From three independent measurements, average values for ΔH -2.45 x 10^3 (±0.24 standard deviation (SD)) kcal.mol^{-1}, ΔS +11.8 (±2.14 SD) cal.K^{-1} mol^{-1}and Kd = 42 (±1.6 SD) µM were obtained. These data are comparable to those reported previously by[18] for intact TolB binding TolA under the same conditions with the exception that here TolB binding is enthalpically driven. These experiments demonstrate that the entire TolA-binding region is contained within the N-terminus of TolB and hence can be used in the form of an isolated peptide. ***b,*** Binding of TolB$^{22-34}$ to the C-terminal domain of ^{15}N-labelled TolA$^{224-347}$ from *P. aeruginosa* followed by changes in ^{1}H, ^{15}N-HSQC spectra, >80% of all TolA peaks were perturbed by TolB$^{22-33}$ binding. Every peak was in slow chemical exchange hence the ratio of the bound and unbound peak volumes corresponds to the ratio of bound/free TolA for each concentration of TolB. *Inset:* shows a portion of a ZZ-exchange HSQC spectrum of a mixture of bound (68%) and free (32%) TolA. The peaks for Ser271 and Gly316 in the bound and free states are labelled; the additional peaks observed arise from exchange between the bound and free states of TolA and confirm the assignment of the bound and free peaks to the same amino acid residue. The titration curves for Ser271, Ser306, Gly316 and Leu347 in TolA were fitted by non-linear regression to obtain the K_d. Average K_d from these fits was 220 (±30 SD) µM (n = 4 residues), which is over five-fold weaker binding than the *E. coli* complex. ***c,*** Fluorescence anisotropy binding curves for *P. aeruginosa* TolB$^{22-33}$ binding the C-terminal domain of TolA. Increasing concentrations of TolA were titrated into 1 µM TolB$^{22-33}$ peptide labelled at its C-terminus (via an additional lysine residue) with FITC. Closed symbols, wild-type TolA$^{224-347}$, K_d = 250 (±10 SD) µM (n = 3), which is in reasonable agreement with the NMR titration data shown in ***b.*** Open symbols, TolA$^{224-344}$ lacking its short C-terminal α-helix, K_d = 86 (±3 SD) µM (n = 3). Deletion of the TolA helix improves TolB binding three-fold.

Supplementary Figure 8

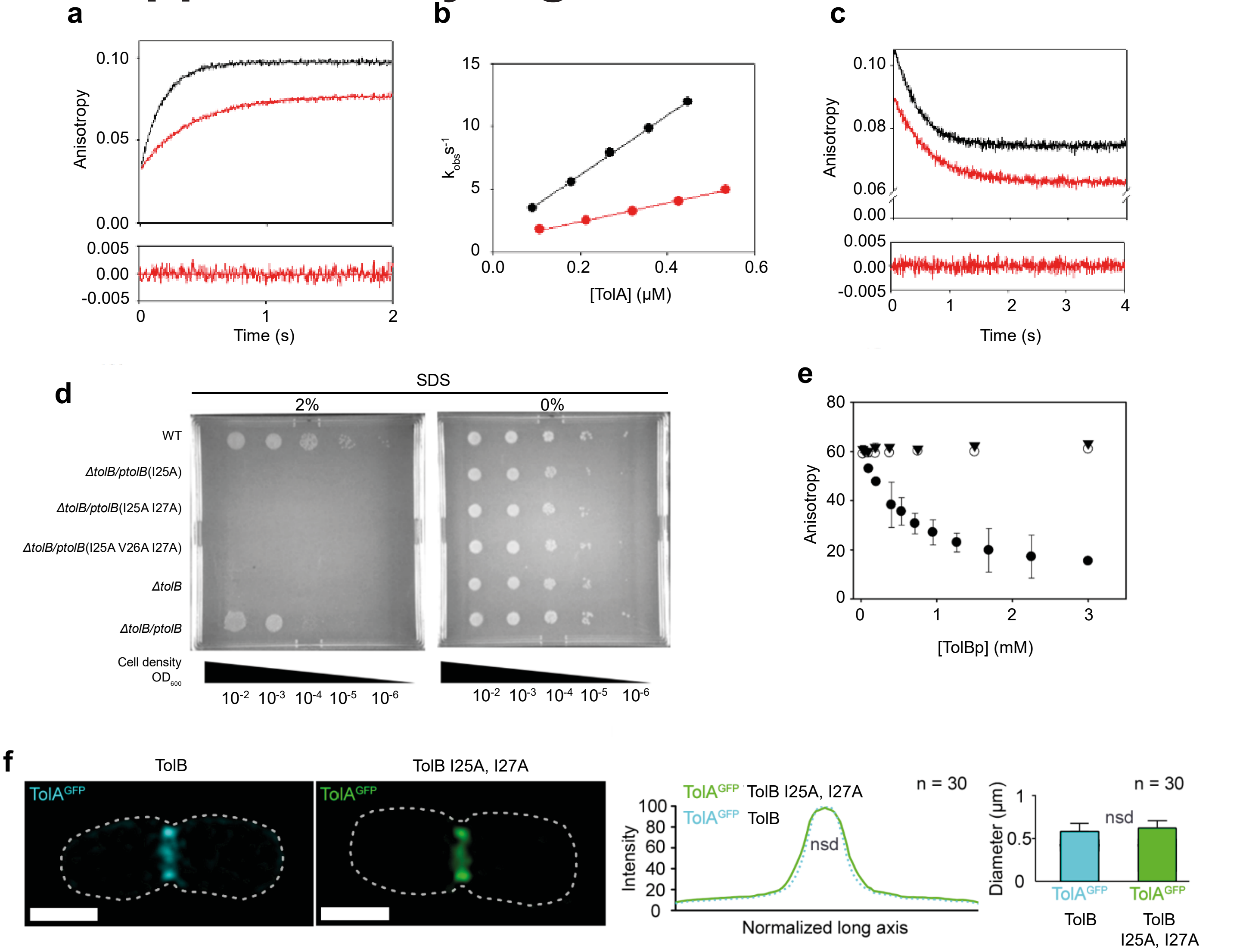

Supplementary Figure 8. Kinetic and thermodynamic measurements of the TolA-TolB interaction and the impact of mutations in vitro and in vivo. All in vitro measurements used the *P. aeruginosa* complex while all in vivo assays used *E. coli*. ***a,*** Pseudo-first-order stopped-flow fluorescence anisotropy traces for *P. aeruginosa* FITC-labelled $TolB^{22\text{-}33}$ binding a 100-fold excess of the C-terminal domain of wild-type TolA (*red*) and TolA with its C-terminal α-helix removed (*black*). Time dependence of the fluorescence polarization of FITC was monitored and fitted to a single exponential equation yielding an observed rate (k_{obs}). Residuals to one such fit are shown below. ***b,*** Association rate constants (k_{on}) for wild-type TolA-$TolB^{22\text{-}33}$ (*red*) and truncated TolA (*black*) were obtained from the concentration dependence of k_{obs} (n = 3; error bars within the data symbols). k_{on} for the wild-type complex was 0.74 x 10^4 (±0.02 SD) $M^{-1}s^{-1}$ while that for the helix truncation was three-fold faster, 2.38 x 10^4 (±0.04 SD) $M^{-1}s^{-1}$, showing that removing the helix speeds up TolB binding. ***c,*** Competition stopped-flow experiments in which FITC-labelled $TolB^{22\text{-}33}$–TolA complexes were chased with a large excess of unlabeled $TolB^{22\text{-}33}$ peptide and monitored by fluorescence anisotropy for wild-type (*red*) and truncated TolA (*black*). k_{off} for the complex changes minimally as a result of truncating the C-terminus of TolA; 0.9 (±0.1 SD) s^{-1} for the wild-type complex and 1.4 (±0.1 SD) s^{-1} for the truncation. ***d,*** Agar plate assay showing the impact of TolB mutations on the stability of the *E. coli* outer membrane in the presence (*left*) or absence (*right*) of 2% SDS. Mutations of the hydrophobic residues at the core of the TolB-TolA complex (TolB Ile25, Val26 or Ile27) produce *tol* phenotypes in *tolB* deletion mutant background that was complemented with plasmid-expressed TolB (p*tolB*). ***e,*** Fluorescence anisotropy competition assay showing that mutations of hydrophobic TolA binding residues in *P. aeruginosa* TolB, Leu25 and Ile27, completely inhibit binding of TolB to TolA. Increasing concentrations of unlabelled competitor peptide were added to FITC-labelled $TolB^{22\text{-}33}$ peptide bound to the C-terminal domain of TolA to which was added and the decrease in anisotropy was monitored for the wild-type complex (*closed spheres*), a TolB L25A mutant peptide (*closed triangles*) and a TolB L25A I27A double mutant peptide (*open spheres*). Data points were obtained in triplicate. ***f,*** SIM data showing that abolition of the TolB-TolA interaction does not affect TolA's recruitment to the divisome. TolA-GFP was imaged in *E. coli* cells in either a wild-type TolB background, *left-hand cell*, or a TolB L25A I27A double mutant, *right-hand cell*, both expressed from a plasmid. Scale bar, 1 μm. Graphical displays beneath the cells show normalized fluorescence distribution on the left-hand panel and the ring diameter of TolA-GFP at the divisome in the right hand panel. Mutation of N-terminal TolB residues have no impact on divisome recruitment of TolA. Mann-Whitney U test and student t test (for intensity distributions and diameter sizes, respectively) show no significant difference between the two conditions (n = 30).

Supplementary Figure 9

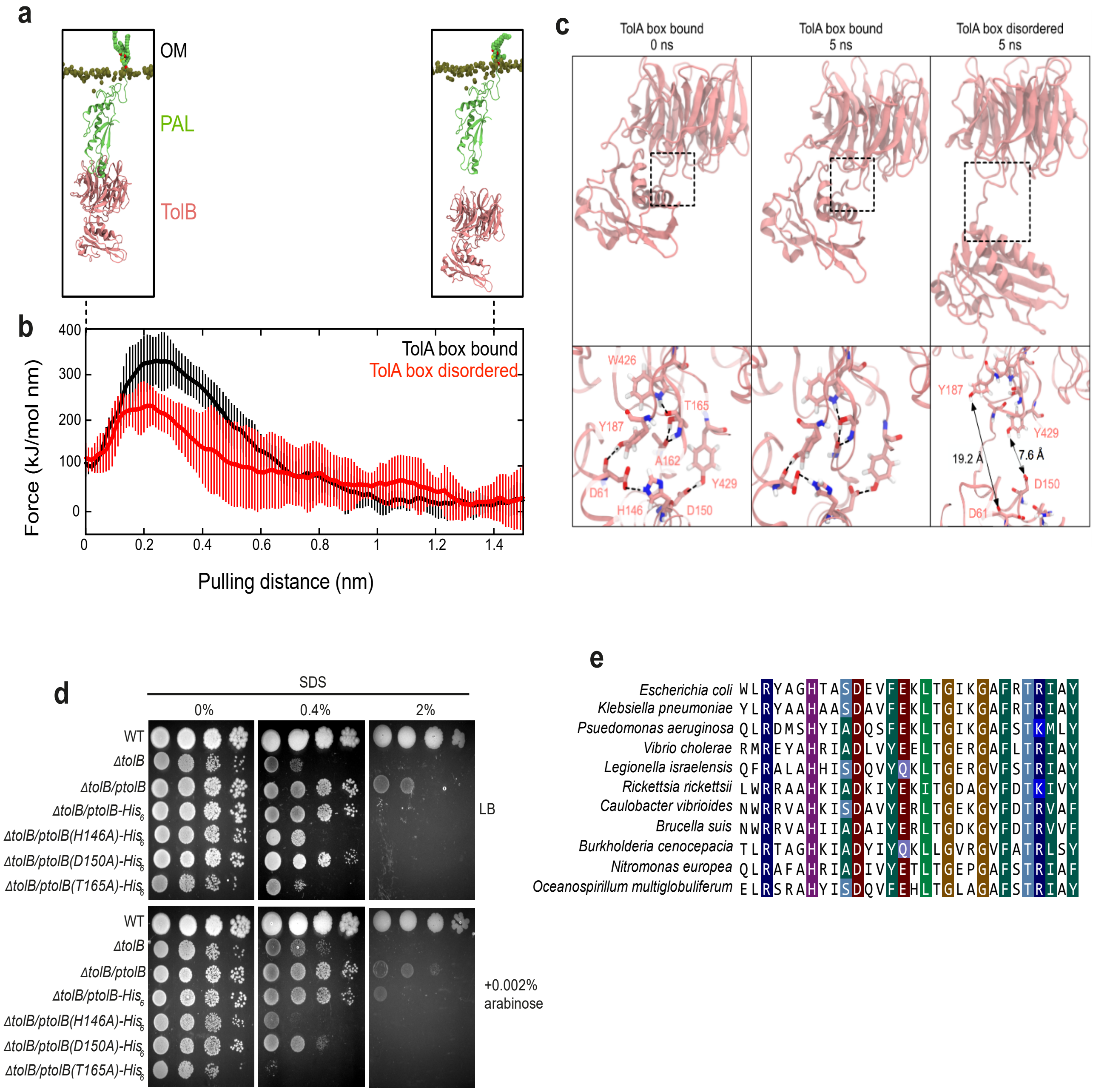

Supplementary Figure 9. Steered molecular dynamics simulations suggest interdomain TolB residues mediate force-dependent dissociation of the TolB-Pal complex. ***a,*** Pal-bound TolB (PDB: 2W8B)[18] was pulled along the z-axis (perpendicular to the plane of the membrane) until it dissociated from Pal. The two images represent the start and end points of one simulation, with bars representing 95% CI. Two conformations of TolB were used in the simulations: the TolA binding region sequestered between TolB's two domains (as in PDB: 2W8B), and the TolA binding region in a disordered state. The pulling force required to detach TolB from Pal in the former conformation was higher than that in the latter conformation. ***b,*** Average pulling force from five independent simulations of the TolB-Pal complex in which the TolB box was ordered (*black*) or disordered (*red*). Error bars indicate the standard deviations. Greater force is needed to dissociate TolB from Pal when its TolA binding region is bound between the two domains of TolB. ***c,*** *Left,* snapshot of TolB at the beginning of one of the TolA box bound simulations. The region shown in dotted square is enlarged beneath the panel. Residues involved in interdomain hydrogen bonds are highlighted and indicated by dotted lines. *Middle,* snapshot at the end of the simulation whereby all the hydrogen bonds were preserved. *Right,* snapshot at the end of one of the simulations where the starting position of the TolA binding region was disordered. Simulated pulling caused the N-terminal domain and the propeller domain to detach from each other. Distances between two pairs of residues that formed hydrogen bonds at the beginning of the simulation are shown. ***d,*** Steered MD simulations identified several inter-domain residues in TolB as potentially mediating force-dependent dissociation from Pal. In particular His146, Asp150 and Thr165. All three residues were mutated individually and expressed off a plasmid in an *E. coli tolB* deletion mutant from an arabinose inducible promoter either in the absence (*left*) or presence of 0.4 or 2% SDS (*right*) and with or without induction with arabinose. Plasmid expressed *tolB* complements the deletion background sufficiently to observe that all three mutations yield tol phenotypes. ***e,*** Clustal W alignment of TolB sequences from Gram-negative bacteria highlighting the conservation of inter-domain residues in α-, β- and γ-proteobacteria.
