# Supplementary material for: The lipoprotein Pal stabilises the bacterial outer membrane during constriction by a mobilisation-and-capture mechanism
Source: Nat Commun. 2020 Mar 11;11:1305. doi: 10.1038/s41467-020-15083-5 (PMC7066135; doi:10.1038/s41467-020-15083-5)
Supplement: Supplementary file 2 — Reporting Summary [file 41467_2020_15083_MOESM2_ESM.pdf]

nature research

Corresponding author(s): Colin Kleanthous

Last updated by author(s): Feb 11, 2020

Reporting Summary

Nature Research wishes to improve the reproducibility of the work that we publish. This form provides structure for consistency and transparency in reporting. For further information on Nature Research policies, see Authors & Referees and the Editorial Policy Checklist .

Statistics

For all statistical analyses, confirm that the following items are present in the figure legend, table legend, main text, or Methods section.

n/a	Confirmed	
☐	☒	The exact sample size (*n*) for each experimental group/condition, given as a discrete number and unit of measurement
☐	☒	A statement on whether measurements were taken from distinct samples or whether the same sample was measured repeatedly
☐	☒	The statistical test(s) used AND whether they are one- or two-sided<br>*Only common tests should be described solely by name; describe more complex techniques in the Methods section.*
☐	☒	A description of all covariates tested
☐	☒	A description of any assumptions or corrections, such as tests of normality and adjustment for multiple comparisons
☐	☒	A full description of the statistical parameters including central tendency (e.g. means) or other basic estimates (e.g. regression coefficient) AND variation (e.g. standard deviation) or associated estimates of uncertainty (e.g. confidence intervals)
☐	☒	For null hypothesis testing, the test statistic (e.g. *F*, *t*, *r*) with confidence intervals, effect sizes, degrees of freedom and *P* value noted<br>*Give P values as exact values whenever suitable.*
☒	☐	For Bayesian analysis, information on the choice of priors and Markov chain Monte Carlo settings
☒	☐	For hierarchical and complex designs, identification of the appropriate level for tests and full reporting of outcomes
☒	☐	Estimates of effect sizes (e.g. Cohen's *d*, Pearson's *r*), indicating how they were calculated

Our web collection on statistics for biologists contains articles on many of the points above.

Software and code

Policy information about availability of computer code

Data collection	Zeiss Zen 2011<br>NimOS 08032018<br>Deltavision softWoRx 6.1.1<br>GeneSys<br>GE/Omega<br>Topspin
Data analysis	FIJI 1.52n<br>Excel 2013<br>GraphPad ver.8.1.2<br>MATLAB<br>ThunderSTORM<br>TrackMate<br>Python (custom scripts)<br>DANGLE<br>PROCHECK-NMR<br>Origin 7.0<br>SigmaPlot 12.0<br>Modeller 9.19

For manuscripts utilizing custom algorithms or software that are central to the research but not yet described in published literature, software must be made available to editors/reviewers. We strongly encourage code deposition in a community repository (e.g. GitHub). See the Nature Research guidelines for submitting code & software for further information.

Data

Policy information about availability of data

All manuscripts must include a data availability statement. This statement should provide the following information, where applicable:

- Accession codes, unique identifiers, or web links for publicly available datasets
- A list of figures that have associated raw data
- A description of any restrictions on data availability

The data supporting the findings of the study are available in the article and its Supporting Information or archived in the PDB or available upon request from the corresponding author.

Field-specific reporting

Please select the one below that is the best fit for your research. If you are not sure, read the appropriate sections before making your selection.

[x] Life sciences [ ] Behavioural & social sciences [ ] Ecological, evolutionary & environmental sciences

For a reference copy of the document with all sections, see nature.com/documents/nr-reporting-summary-flat.pdf

Life sciences study design

All studies must disclose on these points even when the disclosure is negative.

Sample size	For TIRF, SIM and confocal microscopy 30 cells were analysed per strain/condition.
Data exclusions	For single particle analysis (figure 1 b and c) trajectories were eliminated if they occurred outside cell's coordinates. When determining diffusion coefficients from MSD curves, if the determined diffusion coefficients were negative they were discarded.
Replication	All experiments were performed in at least triplicate independent experiments in accordance with well-established reporting procedures for all the assays
Randomization	*Describe how samples/organisms/participants were allocated into experimental groups. If allocation was not random, describe how covariates were controlled OR if this is not relevant to your study, explain why.*
Blinding	The investigators were not blinded to the samples identity.

Reporting for specific materials, systems and methods

We require information from authors about some types of materials, experimental systems and methods used in many studies. Here, indicate whether each material, system or method listed is relevant to your study. If you are not sure if a list item applies to your research, read the appropriate section before selecting a response.

Materials & experimental systems

n/a	Involved in the study	
[ ]	[x]	Antibodies
[x]	[ ]	Eukaryotic cell lines
[x]	[ ]	Palaeontology
[x]	[ ]	Animals and other organisms
[x]	[ ]	Human research participants
[x]	[ ]	Clinical data

Methods

n/a	Involved in the study	
[x]	[ ]	ChIP-seq
[x]	[ ]	Flow cytometry
[x]	[ ]	MRI-based neuroimaging

Antibodies

Antibodies used	Anti-rabbit (Sigma #A6154), Anti-Pal (in house, Bonsor et al., 2009), Anti-TolB (in house, Bonsor et al., 2009)
Validation	Validation data is available at the manufacturer's website
